# Biological ageing, diet quality, and stroke: evidence from two population-based studies and an exploratory hospital-based Chinese cross-sectional dataset

**DOI:** 10.3389/fnut.2026.1895132

**Published:** 2026-09-09

**Authors:** Yiming Liu, Mingming Zhang, Yang Liu

**Affiliations:** 1 Department of Rheumatology and Immunology, The Second Xiangya Hospital, Central South University, Changsha, Hunan, China; 2 Department of Neurosurgery, The Second Xiangya Hospital, Central South University, Changsha, Hunan, China; 3 Department of Rehabilitation Medicine, The Second Xiangya Hospital, Central South University, Changsha, Hunan, China

**Keywords:** biological ageing, change-in-estimate analysis, diet quality, phenoage acceleration, stroke

## Abstract

**Background:**

Diet quality and biological ageing have each been linked to stroke, but whether the association between accelerated biological ageing and stroke reflects poorer diet quality, or is largely independent of it, is unknown. Treating diet quality as an upstream determinant of biological ageing, we examined the association of Phenotypic Age (PhenoAge) acceleration with stroke and how far it changed after adjusting for diet quality.

**Methods:**

We analysed 400,695 UK Biobank participants, 21,754 NHANES adults and 312 adults from a hospital-based dataset (Second Xiangya Hospital). PhenoAge was derived from chronological age and nine biomarkers, and PhenoAge acceleration was PhenoAge minus chronological age. Diet quality was assessed by a Healthy Diet Score (UK Biobank, hospital) and the Healthy Eating Index-2020 (NHANES). Outcomes were incident stroke (record linkage) in the UK Biobank and prevalent stroke in NHANES (self-reported) and the hospital dataset (physician-documented). All models estimated associations; no risk-prediction model was developed or externally validated.

**Results:**

During a median 15.83 years, 11,345 UK Biobank participants developed incident stroke; 729 NHANES and 58 hospital participants had prevalent stroke. Each 1-year increase in PhenoAge acceleration was associated with incident stroke in the UK Biobank (HR 1.060, 95% CI 1.056–1.064) and with prevalent stroke in NHANES (OR 1.058, 1.037–1.080) and the hospital dataset (OR 1.082, 1.002–1.169). In the UK Biobank the association was confined to ischaemic stroke (HR 1.066, 1.061–1.071) but showed no association with haemorrhagic stroke (HR 1.009, 0.998–1.020). Better diet quality was associated with lower PhenoAge acceleration (UK Biobank, high versus low adherence: −0.746 years, −0.811 to −0.681; NHANES: −0.695 years, −0.781 to −0.609). Nevertheless, adding diet quality changed the HR/OR only modestly (11.9, 10.4 and 0.7%; HR 1.060 → 1.053, OR 1.058 → 1.052 and 1.082 → 1.081).

**Conclusion:**

Better diet quality was associated with slower biological ageing, yet accelerated biological ageing was associated with stroke, largely independently of measured diet quality. Because outcome definitions differed and these analyses compare adjusted models rather than decompose a causal pathway, the three estimates are complementary rather than replicative and do not support inferences about prevention or clinical risk stratification.

## 1 Introduction

Stroke remains one of the leading causes of death and disability worldwide. A recent global analysis estimated that, in 2021, there were 93.8 million prevalent stroke survivors, 11.9 million incident strokes, 7.3 million stroke-related deaths, and 160.5 million disability-adjusted life-years attributable to stroke globally (1). Importantly, a substantial proportion of stroke burden is preventable. In the INTERSTROKE study, which included 13,447 patients with acute first stroke and 13,472 controls from 32 countries, ten potentially modifiable risk factors collectively accounted for approximately 90% of the population-attributable risk of stroke, highlighting the importance of prevention strategies targeting modifiable behavioural and metabolic factors (2).

Diet quality is one of the key modifiable factors implicated in stroke prevention. In three large prospective cohorts, compared with participants in the lowest quintile of dietary scores, those in the highest quintile had lower cardiovascular disease risk across several healthy eating patterns, including the Healthy Eating Index-2015, Alternate Mediterranean Diet score, Healthful Plant-Based Diet Index, and Alternative Healthy Eating Index; these dietary scores were also significantly associated with lower stroke risk (3). Evidence from an intervention setting further supports the vascular benefits of healthy dietary patterns. In a randomized trial of 7,447 adults at high cardiovascular risk, assignment to a Mediterranean dietary pattern supplemented with extra-virgin olive oil or nuts was associated with a significantly lower risk of stroke compared with a control diet (4). Similarly, in a prospective analysis of 306,473 UK Biobank participants, an unfavourable lifestyle profile, including poor diet, smoking, obesity, and physical inactivity, was associated with a higher risk of incident stroke compared with a favourable lifestyle profile (5).

Although chronological age is a major determinant of stroke risk, individuals of the same chronological age may differ substantially in physiological function, disease susceptibility, and survival. This heterogeneity has led to increasing interest in biological ageing, which aims to quantify the cumulative deterioration of multiple physiological systems beyond chronological time alone. Phenotypic Age, hereafter referred to as PhenoAge, is a clinically accessible measure of biological ageing derived from chronological age and routinely measured blood biomarkers reflecting inflammation, metabolic function, renal function, immune status, and haematological ageing. In its validation using 11,432 NHANES IV participants, each 1-year increase in PhenoAge was associated with a 9% higher risk of all-cause mortality after accounting for chronological age, supporting its ability to capture clinically meaningful ageing-related risk (6).

Emerging evidence suggests that accelerated biological ageing may be associated with neurological outcomes, including stroke. In a UK Biobank analysis of 325,870 participants without neurological disease at baseline, clinical biomarker-based biological ageing measures were associated with subsequent neurological disorders. During a mean follow-up of 9.0 years, 2,515 incident ischaemic strokes were documented, and higher biological age was associated with greater ischaemic stroke risk, with one standard deviation higher PhenoAge-related biological age associated with increased future ischaemic stroke risk (7). More recently, a prospective cohort study of 253,932 UK Biobank participants reported 5,460 incident strokes during a median follow-up of 13.6 years. In that study, each standard deviation increase in PhenoAge acceleration was associated with a higher risk of total stroke, ischaemic stroke, intracerebral haemorrhage, and subarachnoid haemorrhage (8). These findings suggest that biological ageing may provide information on stroke susceptibility beyond chronological age.

Diet quality may be closely linked to biological ageing. In a case-cohort sample of 2,694 women, higher adherence to healthy dietary patterns was inversely associated with DNA methylation-based age acceleration. The strongest association was observed for the Alternative Healthy Eating Index-2010, where each 1-standard deviation increase in diet quality was associated with lower PhenoAge acceleration and lower GrimAge acceleration (9). In another analysis of 16,666 adults from NHANES 1999–2018, healthy dietary patterns were associated with biological age deceleration, whereas higher dietary inflammatory index scores were associated with greater odds of accelerated biological ageing; compared with the lowest dietary inflammatory index category, the highest category was associated with accelerated PhenoAge (10). These findings support a potential link between diet quality and ageing biology.

Previous studies have primarily examined biological ageing as a risk marker for stroke or as a potential mediator linking overall healthy behaviours with stroke risk. Few studies have quantified how far the association between accelerated biological ageing and stroke changes after additional adjustment for diet quality, and evidence integrating a large prospective UK cohort, a nationally representative US population, and a hospital-based Chinese population remains limited. Clarifying this relationship may help determine whether diet quality contributes to the observed association between biological ageing and stroke across different study settings. Therefore, using data from the UK Biobank, NHANES, and the Second Xiangya Hospital, we examined the association of PhenoAge acceleration with stroke, prospectively for incident stroke in the UK Biobank and cross-sectionally for prevalent stroke in the two remaining datasets, and assessed how far this association changed after additional adjustment for diet quality. We further evaluated whether the association between biological ageing and stroke differed across population subgroups. Because diet quality would ordinarily be expected to influence biological ageing rather than to lie downstream of it, we specified the causal structure in advance: diet quality is treated as an upstream determinant of PhenoAge acceleration and as a shared antecedent of stroke, not as a mediator of the biological ageing–stroke association. UK Biobank has already been used to relate clinical biomarker-based biological ageing to stroke (7, 8); the present analytic sample overlaps substantially with those reports, and the incremental contribution here is not to re-demonstrate that association but to quantify explicitly, and in parallel across a prospective cohort, a nationally representative survey and an exploratory clinical sample, how little of the estimated association is displaced when diet quality is added to the adjustment set.

## 2 Methodology

### 2.1 Study population

This study used data from three sources: the UK Biobank, the US National Health and Nutrition Examination Survey (NHANES), and a hospital-based cross-sectional dataset from the Second Xiangya Hospital, Central South University. The UK Biobank is a large prospective cohort study that recruited more than 500,000 participants aged 40–69 years between 2006 and 2010. At baseline, participants completed touchscreen questionnaires on sociodemographic characteristics, socioeconomic status, lifestyle factors, dietary habits, sleep behaviours, and medical history, underwent physical measurements, and provided biological samples. Health outcomes were ascertained through linkage to hospital admission records and death registry data. NHANES is a nationally representative survey of the civilian, non-institutionalized US population. Participants were selected using a complex, multistage probability sampling design and underwent household interviews, physical examinations, dietary assessments, and laboratory testing at mobile examination centres. In the present study, adult participants from NHANES with available information on biological ageing biomarkers, dietary assessment, stroke status, and relevant covariates were included. The Second Xiangya Hospital dataset was derived from adult patients who underwent clinical evaluation at the Second Xiangya Hospital, Central South University. A hospital-based cross-sectional design was used. The present analysis is a retrospective secondary analysis of de-identified records held in an existing hospital clinical database; no participants were recruited for this study. Participants with and without stroke had been enrolled consecutively into that database over a single defined period (1 January 2022 to 31 December 2022), from the same clinical services and through the same process, so that the comparison group was drawn from the source population that generated the stroke cases (11). Patients attended primarily for routine health checks and for the management of chronic disease. Eligible participants were adults aged 18 years or older with available information on stroke status, sociodemographic characteristics, lifestyle factors, dietary assessment, anthropometric measurements, medical history, and laboratory biomarkers required for PhenoAge calculation. Clinical and laboratory data were obtained from routine electronic medical records, physical examination records, blood-test results, and imaging reports at the index clinical encounter. Stroke status was determined according to physician diagnosis and was supported, where applicable, by neurological evaluation and neuroimaging findings, including computed tomography or magnetic resonance imaging. Participants without a recorded diagnosis or history of stroke at the index assessment were classified as non-stroke controls. Stroke-free status was confirmed by review of the complete clinical record, including neurological history and available neuroimaging. Because C-reactive protein, white blood cell count, glucose, albumin and creatinine are all components of PhenoAge and are all altered by the acute-phase response that follows stroke, a raised PhenoAge measured during an acute admission could be a consequence of the event rather than a marker of pre-existing accelerated ageing (12). Laboratory measurements used to derive PhenoAge were therefore taken outside the acute phase, and the interval between the most recent stroke and blood sampling was recorded for every participant with stroke. Among the 58 participants with stroke, none presented with acute stroke, 15 had recurrent stroke and 43 had chronic disease or a past medical history of stroke. The median interval between stroke onset and blood sampling was 450 days (interquartile range 180–800 days), and at the time of sampling no participant was in the acute phase, 10% were in the rehabilitation phase and 90% were clinically stable.

**Figure 1:**
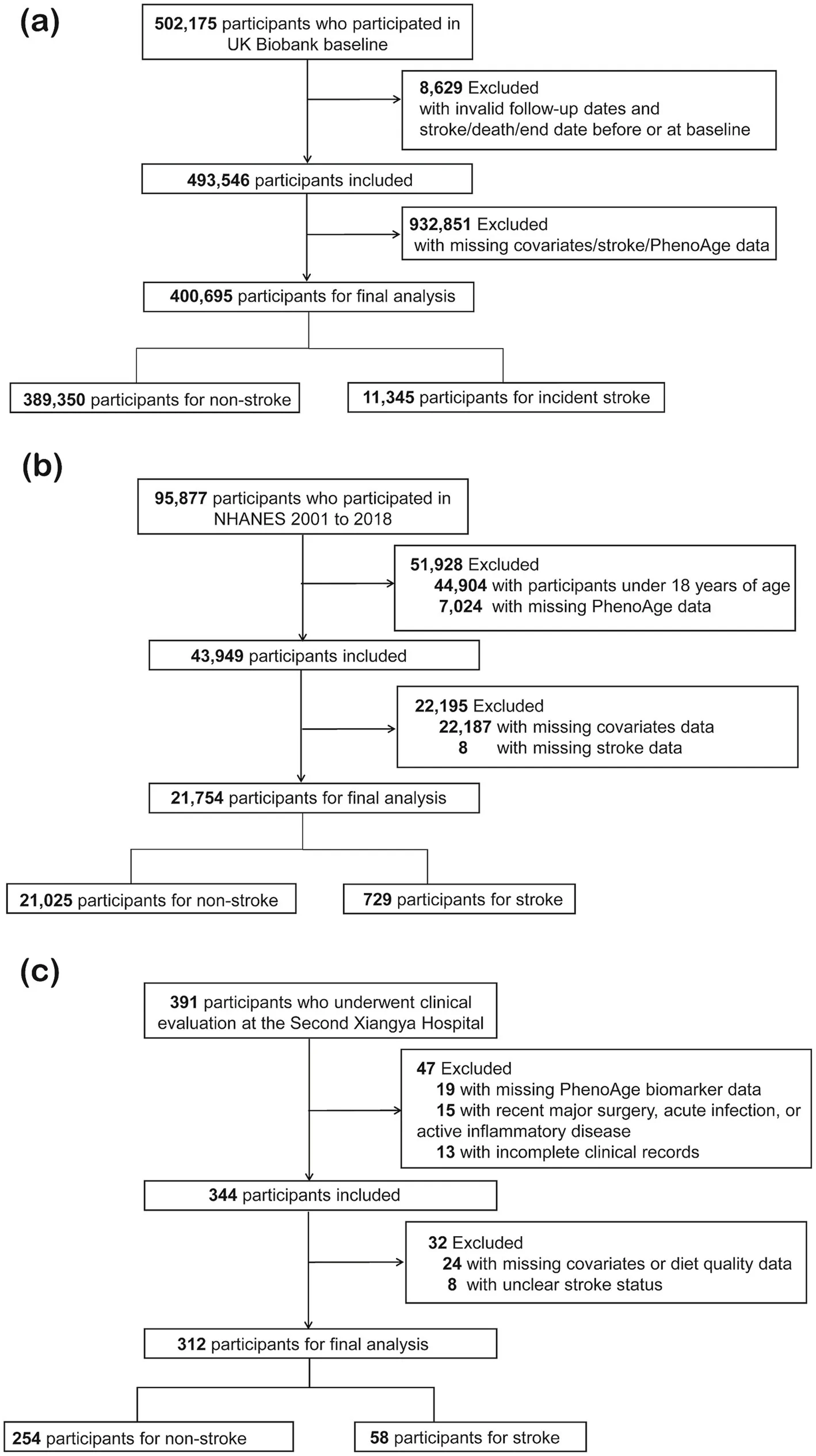
Flowchart of participant selection. **(a)** Shows the selection of participants from the UK Biobank cohort, resulting in 400,695 participants for the final analysis, including 389,350 participants without stroke and 11,345 participants with incident stroke. **(b)** Shows the selection of participants from NHANES 2001–2018, resulting in 21,754 participants for the final analysis, including 21,025 participants without stroke and 729 participants with self-reported physician-diagnosed stroke. Panel **(c)** Shows the selection of participants from the Second Xiangya Hospital cross-sectional dataset, resulting in 312 participants for the final analysis, including 254 participants without stroke and 58 participants with stroke.

To ensure comparability with the population-based datasets and to reduce distortion of ageing-related biomarkers, participants were excluded if they had missing data on PhenoAge biomarkers, diet quality, stroke status, or key covariates. Additional exclusions were applied for individuals with recent major surgery, acute infection, active inflammatory disease, severe haematological disease, or other acute clinical conditions that could substantially influence inflammatory, metabolic, renal, or haematological biomarkers at the time of assessment. After applying the inclusion and exclusion criteria, 312 participants from the Second Xiangya Hospital were included in the final analysis, including 254 participants without stroke and 58 participants with stroke. In the UK Biobank, participants were eligible if they had available information on clinical biomarkers required for PhenoAge calculation, diet quality, stroke status, and relevant covariates. Participants with missing information on key exposure, diet quality, outcome, or covariates were excluded. The final UK Biobank analytic sample included 400,695 participants, among whom 389,350 remained free of stroke and 11,345 developed stroke during follow-up. For NHANES, the final analytic sample included 21,754 participants, among whom 21,025 did not report stroke and 729 reported a history of stroke. See Figure 1 for the detailed screening process.

### 2.2 Exposure: biological ageing

Biological ageing was estimated using the Phenotypic Age algorithm, hereafter referred to as PhenoAge (13). PhenoAge is a composite biological ageing measure derived from chronological age and nine routinely measured clinical biomarkers: serum albumin, creatinine, glucose, C-reactive protein, lymphocyte percentage, mean corpuscular volume, red cell distribution width, alkaline phosphatase, and white blood cell count (14, 15). These biomarkers reflect multiple physiological domains, including metabolic function, renal function, systemic inflammation, immune status, and haematological ageing (13, 15).

PhenoAge was calculated using the previously validated algorithm proposed by Levine et al. (16). Briefly, a linear predictor was first derived from chronological age and the nine clinical biomarkers. This linear predictor was then converted into a mortality score and subsequently transformed into PhenoAge (17). To ensure reproducibility, biomarker units were harmonized to match those required by the original algorithm before the linear predictor was computed: albumin in g/L, creatinine in μmol/L, glucose in mmol/L, C-reactive protein in mg/dL and entered after natural-logarithmic transformation, lymphocyte percentage in %, mean corpuscular volume in fL, red cell distribution width in %, alkaline phosphatase in U/L, white blood cell count in 10^3^ cells/μL and chronological age in years. Values reported in other units in NHANES or in the hospital laboratory system were converted to these units before calculation. Biologically implausible values were set to missing, and remaining values were winsorised at the 0.1st and 99.9th percentiles within each dataset so that extreme laboratory results could not dominate the composite score.

The formula for PhenoAge calculation was as follows:

$$\begin{array}{l} \mathrm{xb}=-19.9067-0.0336\times \text{albumin}\;(g/L)+0.0095\times \text{creatinine}\;(\mu \mathrm{mol}/L)\\ +0.1953\times \text{glucose}\;(\text{mmol}/L)+0.0954\times \ln[C-\text{reactive protein}\;(\mathrm{mg}/\mathrm{dL})]\\ -0.0120\times \text{lymphocyte percentage}\;(\%)+0.0268\\ \times \text{mean corpuscular volume}\;(\mathrm{fL})+0.3306\times \mathrm{red}\;\text{cell distribution width}\;(\%)\\ +0.00188\times \text{alkaline phosphatase}\;(U/L)+0.0554\\ \times \text{white blood cell count}\;(10^{3}\;\text{cells}/\mathrm{\mu L})+0.0804\times \text{chronological}\;\mathrm{age}\;(\text{years}). \end{array}$$

γ=0.0076927.

Mortality risk=1−exp{−exp(xb)×[exp(120×γ)−1]/γ}.

$$\begin{array}{c} \text{PhenoAge}=141.50225\\ +\ln\{-0.00553\times \ln(1-\text{Mortality risk})\}/0.090165. \end{array}$$

To quantify accelerated biological ageing, PhenoAge acceleration was calculated as the difference between PhenoAge and chronological age:

PhenoAge acceleration=PhenoAge−chronologicalage.

A positive value indicated that an individual was biologically older than their chronological age, whereas a value of zero or below indicated that an individual was biologically younger than or comparable to their chronological age. Accordingly, participants with PhenoAge acceleration greater than 0 were classified as biologically older, and those with PhenoAge acceleration less than or equal to 0 were classified as biologically younger. PhenoAge acceleration was analysed both as a continuous variable, per 1-year increase, and as a categorical variable, including biologically younger versus biologically older groups and quartiles of PhenoAge acceleration (18). Because chronological age is itself a term in the PhenoAge algorithm, the difference score may retain a residual correlation with chronological age, which is also adjusted for in the regression models. We therefore report the Pearson correlation of chronological age with PhenoAge and with PhenoAge acceleration in each dataset, together with variance inflation factors for all terms in the primary models. The difference-score definition was retained as the primary specification because it preserves a directly interpretable deviation expressed in years and was the form used in our original analytic specification. We state explicitly that this is not the definition used in the original derivation of PhenoAge acceleration, which was obtained as the residual from a linear regression of PhenoAge on chronological age, nor in the recent UK Biobank analysis of biological ageing and stroke, which used the same residual-based approach (6, 8), which was evaluated as a sensitivity analysis. As a prespecified sensitivity analysis, PhenoAge acceleration was redefined as the residual from a linear regression of PhenoAge on chronological age fitted separately within each dataset, a quantity that is orthogonal to chronological age by construction (19); in a further sensitivity analysis chronological age was modelled flexibly with a restricted cubic spline (three knots at the 10th, 50th and 90th percentiles) rather than as a linear term. The threshold of greater than zero was retained because it carries a direct interpretation, namely that measured biological age exceeds chronological age. The continuous analysis is treated as primary. To allow comparison across datasets with different exposure distributions, and to separate statistical significance from clinical magnitude, effect estimates are additionally reported per one standard deviation and, in the UK Biobank, per five-year increase in PhenoAge acceleration.

### 2.3 Outcome: stroke

In the UK Biobank, incident stroke events were identified through linked hospital admission records and death registry data. Participants with evidence of stroke before or at baseline, death before or at baseline, or invalid follow-up information were excluded before outcome ascertainment. Stroke was defined according to International Classification of Diseases, 10th Revision (ICD-10) codes recorded in hospital inpatient records or death registries. The primary outcome was total stroke, including subarachnoid haemorrhage (I60), intracerebral haemorrhage (I61), other non-traumatic intracranial haemorrhage (I62), cerebral infarction or ischaemic stroke (I63), and stroke not specified as haemorrhage or infarction (I64). Stroke events were identified using diagnosis codes from hospital inpatient records and underlying or contributory causes of death from death registry data. Participants were followed from baseline assessment until the first recorded stroke event, death, loss to follow-up, or the end of available record linkage, whichever occurred first. In NHANES, stroke status was assessed using self-reported physician diagnosis. Participants were asked whether a doctor or other health professional had ever told them that they had a stroke. Participants who answered “yes” were classified as having a history of stroke, whereas those who answered “no” were classified as not having stroke. Participants with uncertain, refused, or missing responses were excluded. Because NHANES is cross-sectional and stroke was self-reported, the timing and subtype of stroke could not be determined. In the Second Xiangya Hospital dataset, stroke status was determined at the index clinical encounter using information from electronic medical records, discharge diagnoses, neurological evaluation records, and neuroimaging reports. Stroke was defined according to physician-documented diagnosis and was supported, where available, by brain computed tomography or magnetic resonance imaging findings. Consistent with the UK Biobank outcome definition, total stroke included haemorrhagic stroke, ischaemic stroke, and stroke not otherwise specified. Participants with a recorded diagnosis or documented history of stroke were classified as having stroke, whereas those without evidence of stroke in the available clinical records were classified as non-stroke. Participants with unclear, inconsistent, or unavailable stroke-status information were excluded. Because the Second Xiangya Hospital dataset had a cross-sectional design, stroke status was analysed as prevalent stroke at the time of clinical assessment, and the temporal sequence between biological ageing indicators and stroke could not be established. Because total stroke aggregates clinically heterogeneous entities with different pathophysiology and different relationships to ageing and diet, ischaemic stroke (I63) and haemorrhagic stroke (I60–I62) were also analysed separately in the UK Biobank, where the number of events was sufficient to support subtype-specific models. Hospital inpatient linkage in the UK Biobank was complete to 31 October 2022 in England, 31 July 2021 in Scotland and 28 February 2018 in Wales; follow-up was therefore censored at the nation-specific linkage end date, and a sensitivity analysis applied the earliest of these dates to the whole cohort so that follow-up duration could not differ systematically by country of residence.

### 2.4 Confounders

Potential confounders were selected using an explicit causal framework rather than biological plausibility alone; the assumed structure, including the position of diet quality as an upstream determinant of biological ageing, is presented as a directed acyclic graph in Supplementary Figure S1 (20). Because the three data sources differed in study design and variable availability, covariates were harmonized as much as possible and included in dataset-specific models where available. Core covariates included age, sex, educational level, body mass index, comorbidity index, smoking status, alcohol consumption, sleep duration, and income or cohort-specific socioeconomic indicators. Age was analysed as a continuous variable. Sex was categorized as male or female. Race was available in the UK Biobank and NHANES and was categorized as White or non-White, whereas race was not evaluated in the Second Xiangya Hospital dataset. Socioeconomic deprivation was assessed using the Townsend Deprivation Index in the UK Biobank and the poverty-income ratio in NHANES. In the UK Biobank, participants were classified as deprived or non-deprived according to the Townsend Deprivation Index (TDI), with higher positive scores indicating greater material deprivation and negative scores indicating greater affluence. In NHANES, socioeconomic status was assessed using the poverty-income ratio (PIR), with lower PIR indicating greater socioeconomic disadvantage. A comparable deprivation or PIR variable was not available in the Second Xiangya Hospital study; therefore, socioeconomic status in this dataset was represented by income level only. Educational level was categorized as higher, upper secondary, lower secondary, and other or unknown. Body mass index was calculated as weight in kilograms divided by height in metres squared and was analysed both as a continuous variable and as a categorical variable. BMI categories were defined as underweight, BMI < 18.5 kg/m^2^; normal weight, 18.5 ≤ BMI < 25 kg/m^2^; overweight, 25 ≤ BMI < 30 kg/m^2^; and obesity, BMI ≥ 30 kg/m^2^. Comorbidity burden was assessed using the comorbidity index and analysed as a continuous variable. See Supplementary material S1 and Supplementary Tables S1, S2 for the detailed evaluation methods and coding rules. Smoking status was assessed as ever smoked cigarettes and categorized as never smoking versus ever smoking. Alcohol consumption was categorized as never, previous, current, and unknown. Sleep duration was assessed using self-reported average sleep hours per day and analysed as a continuous variable. Sleep duration was also categorized into optimal sleep, short sleep, and long sleep groups according to predefined cut-points. Income was categorized into low, middle, and high groups in the UK Biobank and the Second Xiangya Hospital dataset. In NHANES, socioeconomic level was further categorized into low, middle, and high groups according to the poverty-income ratio. Residence was available in the UK Biobank and the Second Xiangya Hospital dataset and was categorized as urban, rural, or unknown. A comparable residence variable was not included in the NHANES analyses. Further detailed classification and explanation of these confounders can be found in Supplementary material S2 and Supplementary Tables S2, S3. Several conditions contained in the comorbidity index, in particular hypertension, diabetes and atrial fibrillation, are major causes of stroke but may also be downstream clinical manifestations of accelerated biological ageing, and glucose, creatinine, C-reactive protein and albumin are themselves PhenoAge components; simultaneous adjustment for the full comorbidity score therefore risks removing part of the pathway of interest (21). Conversely, an unweighted count gives equal weight to conditions with very different stroke relevance and may leave major determinants inadequately controlled. We report a prespecified sequence of nested models: Model 1 adjusted for chronological age and sex; Model 2 additionally adjusted for sociodemographic and lifestyle variables; Model 3 additionally adjusted for comorbidity burden, modelled as the composite comorbidity index; and Model 4 additionally adjusted for diet quality. A further sensitivity analysis excluded potentially downstream comorbidities from the adjustment set altogether. Individual vascular diagnoses and medication records were not available as separate variables across all three datasets, so vascular comorbidity was represented by the composite index.

### 2.5 Diet quality

Diet quality was assessed using study-specific dietary indices and was examined as a diet-quality indicator in change-in-estimate analyses of the association between biological ageing and stroke. In the UK Biobank, diet quality was assessed using a composite Healthy Diet Score ranging from 0 to 5 (22, 23). One point was assigned for each of the following five dietary criteria met: fruit intake of at least 3 servings per day; vegetable intake of at least 3 servings per day; fish consumption, including oily and/or non-oily fish, of at least 2 servings per week; processed meat intake of no more than 1 serving per week; and unprocessed red meat intake of no more than 1–2 servings per week. The total Healthy Diet Score was calculated by summing the five components. Participants were then categorized into three healthy-diet adherence groups: low adherence, defined as 0–1 points; moderate adherence, defined as 2–3 points; and high adherence, defined as 4–5 points. Higher adherence indicated greater conformity to a healthy dietary pattern. In the NHANES study, diet quality was assessed using the Healthy Eating Index-2020, which measures adherence to the 2020–2025 Dietary Guidelines for Americans (24). The total HEI-2020 score ranges from 0 to 100, with higher scores indicating better adherence to recommended dietary patterns. The HEI-2020 includes 13 components, consisting of nine adequacy components and four moderation components. The adequacy components include total fruits, whole fruits, total vegetables, greens and beans, whole grains, dairy, total protein foods, seafood and plant proteins, and fatty acids. The moderation components include refined grains, sodium, added sugars, and saturated fats. Component scores are density-based, generally calculated per 1,000 kcal, except for fatty acids, which are calculated as the ratio of polyunsaturated and monounsaturated fatty acids to saturated fatty acids. The total HEI-2020 score was calculated as the sum of all component scores. Participants were categorized into three dietary-adherence groups using *a priori* cut-points: low adherence, defined as 0–40 points; moderate adherence, defined as 41–60 points; and high adherence, defined as 61–100 points. In the Second Xiangya Hospital dataset, diet quality was assessed at the index clinical encounter using a structured dietary questionnaire that recorded usual intake of major food groups. To improve comparability with the UK Biobank analysis, a five-component Healthy Diet Score was constructed using the same dietary domains, including fruit intake, vegetable intake, fish intake, processed meat intake, and unprocessed red meat intake. One point was assigned for each healthy dietary criterion met, and the total score ranged from 0 to 5. Participants were categorized as having low, moderate, or high healthy-diet adherence using the same cut-points as those applied in the UK Biobank: 0–1, 2–3, and 4–5 points, respectively. In all datasets, higher healthy-diet adherence indicated better overall diet quality. Because dietary information in NHANES and the Second Xiangya Hospital dataset was obtained at a single assessment, diet quality could not be established as a temporally confirmed exposure preceding stroke. In NHANES it was therefore interpreted as a concurrent dietary indicator. In the Second Xiangya Hospital dataset the questionnaire asked participants with stroke to report habitual intake during the 12 months preceding the stroke event, so the score represents retrospectively reported pre-stroke habitual diet collected at the index cross-sectional assessment; this reduces, but does not remove, the possibility that reporting was influenced by dietary change after the event, and recall bias cannot be excluded. For NHANES, the analysis included the 2001–2018 cycles, and HEI-2020 was calculated with the simple HEI scoring algorithm applied to the first 24-h dietary recall using the day-1 dietary recall weight, with all component scores expressed per 1,000 kcal except the fatty-acid component, which is a ratio; total energy intake was therefore accounted for through density-based scoring rather than by separate energy adjustment (25). Because a single 24-h recall reflects intake on one day, episodically consumed foods such as fish and whole grains are captured imperfectly and within-person day-to-day variation is not removed, so individual scores are shrunk toward the population mean. For the hospital dataset, the complete dietary questionnaire, its response options, reference period, scoring rules and mode of administration are provided in Supplementary material S8. We emphasise that the three instruments are not equivalent: HEI-2020 comprises 13 energy-density-adjusted components derived from quantitative recalls, whereas both five-component scores are constructed from a small number of food-frequency items, and the instruments differ further in cultural relevance and in measurement error. Categorising each score into low, moderate and high adherence aligns their presentation but does not make them measures of the same construct, and agreement in the direction of association across them should therefore not be interpreted as formal replication.

### 2.6 Statistical analyses

Baseline characteristics were summarized according to stroke status in the UK Biobank, NHANES, and the Second Xiangya Hospital dataset. Continuous variables were presented as means and standard deviations, and categorical variables were presented as frequencies and percentages. Differences between participants with and without stroke were assessed using t tests or Wilcoxon rank-sum tests for continuous variables, as appropriate, and chi-square tests or Fisher’s exact tests for categorical variables, as appropriate. For the UK Biobank cohort study, Cox proportional hazards regression models were used to estimate hazard ratios (HRs) and 95% confidence intervals (CIs) for the association between biological ageing and incident stroke. Participants were followed from baseline assessment until the first diagnosis of stroke, death, loss to follow-up, or the end of follow-up, whichever occurred first. PhenoAge acceleration was analysed as a continuous variable per 1-year increase, as a binary variable comparing biologically older with biologically younger participants, and as quartiles. For the NHANES cross-sectional study, survey-weighted multivariable logistic regression models were used to estimate odds ratios (ORs) and 95% CIs for the association between biological ageing and self-reported physician-diagnosed stroke status. Sampling weights, strata, and primary sampling units were incorporated in all NHANES analyses to account for the complex multistage survey design and to obtain nationally representative estimates. For analyses combining multiple NHANES cycles, survey weights were adjusted according to NHANES analytic guidelines. Weight selection was based on the most restrictive analytic component included in each analysis, including laboratory biomarkers for PhenoAge calculation and 24-h dietary recall data for diet-quality assessment. For the Second Xiangya Hospital cross-sectional dataset, multivariable logistic regression models were used to estimate ORs and 95% CIs for the association between biological ageing and physician-diagnosed stroke status at the index clinical encounter. The primary UK Biobank models were adjusted for age, sex, race, educational level, BMI category, comorbidity index, smoking status, alcohol consumption, sleep-duration group, Townsend Deprivation Index, income level, and residence. The primary NHANES models were adjusted for age, sex, race, educational level, BMI category, comorbidity index, smoking status, alcohol consumption, sleep-duration group, and poverty-income ratio. The primary Second Xiangya Hospital models were adjusted for age, sex, educational level, BMI category, comorbidity index, smoking status, alcohol consumption, sleep-duration group, income level, and residence. Race and deprivation or poverty-income ratio were not included in the Second Xiangya Hospital models because these variables were not evaluated in this dataset. Diet quality was not included in the base model. A diet-adjusted model additionally included healthy-diet adherence to examine the extent to which the association between PhenoAge acceleration and stroke was attenuated after accounting for diet quality. Restricted cubic spline models with four knots were used to examine potential non-linear associations of PhenoAge acceleration with stroke. Non-linearity was assessed using likelihood ratio tests comparing models with and without spline terms. All NHANES descriptive statistics, including the means, proportions and *p* values presented in Table 1, were calculated with the survey weights, strata and primary sampling units, using design-based Wald tests for continuous variables and Rao–Scott chi-square tests for categorical variables, so that the complex sampling design is reflected in the descriptive analyses and not only in the regression models. Participants were screened across the 2001–2018 NHANES cycles. Because C-reactive protein was not measured in the 2011–2012 and 2013–2014 cycles, and the PhenoAge algorithm requires all nine biomarkers, these two cycles contributed no complete cases; the final complete-case analysis therefore drew on seven survey cycles. The day-1 dietary recall weight was accordingly divided by seven, in accordance with the NHANES analytic guidelines for combining discontinuous survey cycles (26), and singleton strata were centred at the overall sample mean. Age-standardised prevalence estimates were not calculated; the NHANES prevalence estimates presented in Table 1 are survey-weighted crude estimates. Standardised mean differences are additionally reported for the UK Biobank given the large sample size (27). For the UK Biobank, the proportional-hazards assumption was tested formally using scaled Schoenfeld residuals, and the global test statistic together with the exposure-specific statistic is reported rather than merely stating that the assumption was examined. The four spline knots were placed at the 5th, 35th, 65th and 95th percentiles of the exposure distribution. Incidence rates per 1,000 person-years are reported alongside hazard ratios. Death is a competing event for stroke and is itself strongly related to accelerated biological ageing. In the cause-specific framework, censoring at the time of death is the appropriate treatment of the competing event, so the primary Cox model already estimates the cause-specific hazard; to complement it on the cumulative-incidence scale we additionally fitted a Fine–Gray subdistribution hazard model with death before stroke as the competing event (28).

**Table 1:** Baseline characteristics of participants according to stroke status in the UK Biobank (*n* = 400,695), NHANES (*n* = 21,754), and Second Xiangya Hospital (*n* = 312).

Characteristics	UK Biobank			NHANES			Second Xiangya Hospital		
	Non-stroke (*n* = 389,350)	Stroke (*n* = 11,345)	*p*-value (SMD)	Non-stroke (*n* = 21,025)	Stroke (*n* = 729)	*P*-value	Non-stroke (*n* = 254)	Stroke (*n* = 58)	*P*-value
Age, years	56.02 (8.06)	60.09 (7.29)	<0.001 (0.531)	45.82 (0.35)	66.14 (0.68)	<0.001	57.75 (12.73)	66.34 (10.62)	<0.001
Male, %	182,398 (46.8)	6,409 (56.5)	<0.001 (0.194)	48.8	46.2	0.215	127 (50.0)	35 (60.3)	0.155
White race, %	370,548 (95.2)	10,859 (95.7)	0.007 (0.024)	68.5	71.2	0.182	-	-	-
Deprivation/low PIR, %	107,398 (27.6)	3,452 (30.4)	<0.001 (0.061)	15.2	24.8	<0.001	-	-	-
Educational level, %			<0.001 (0.115)			<0.001			0.020
Higher	183,366 (47.1)	4,757 (41.9)		59.1	41.3		78 (30.7)	9 (15.5)	
Upper secondary	128,607 (33.0)	3,495 (30.8)		25.4	28.5		85 (33.5)	16 (27.6)	
Lower secondary	75,907 (19.5)	3,028 (26.7)		14.8	29.4		87 (34.3)	31 (53.4)	
Other/unknown	1,470 (0.4)	65 (0.6)		0.7	0.8		4 (1.6)	2 (3.4)	
BMI, kg/m^2^	27.35 (4.73)	27.98 (4.81)	<0.001 (0.131)	28.61 (0.12)	30.15 (0.34)	<0.001	25.71 (3.68)	26.70 (3.88)	0.080
BMI, %			<0.001 (0.188)			<0.001			0.112
Underweight	2,725 (0.7)	68 (0.6)		1.8	1.2		8 (3.1)	1 (1.7)	
Normal weight	127,698 (32.8)	2,916 (25.7)		29.5	20.8		90 (35.4)	14 (24.1)	
Overweight	167,421 (43.0)	4,765 (42.0)		33.2	34.5		100 (39.4)	22 (37.9)	
Obesity	91,506 (23.5)	3,596 (31.7)		35.5	43.5		56 (22.0)	21 (36.2)	
Comorbidity index	1.05 (1.32)	1.70 (1.71)	<0.001 (0.421)	0.58 (0.02)	1.35 (0.05)	<0.001	0.89 (0.78)	1.45 (0.93)	<0.001
Ever smoked cigarettes, %	174,876 (44.9)	5,991 (52.8)	<0.001 (0.158)	45.2	62.8	<0.001	91 (35.8)	31 (53.4)	0.013
Alcohol drink, %			<0.001 (0.082)			<0.001			0.005
Never	14,374 (3.7)	502 (4.4)		12.5	15.4		40 (15.7)	15 (25.9)	
Previous	12,802 (3.3)	496 (4.4)		15.8	28.2		28 (11.0)	13 (22.4)	
Current	361,960 (93.0)	10,340 (91.1)		71.7	56.4		186 (73.2)	30 (51.7)	
Unknown	214 (0.1)	7 (0.1)		0.0	0.0		0 (0.0)	0 (0.0)	
Sleep duration, hours	7.15 (1.07)	7.19 (1.19)	<0.001 (0.035)	7.05 (0.03)	7.12 (0.08)	0.384	7.01 (1.20)	7.28 (1.35)	0.165
Sleep duration group, %			<0.001 (0.054)			0.012			0.020
Optimal sleep	289,539 (74.4)	8,181 (72.1)		63.5	55.2		169 (66.5)	31 (53.4)	
Short	93,640 (24.1)	2,883 (25.4)		31.2	34.5		71 (28.0)	18 (31.0)	
Long	6,171 (1.6)	281 (2.5)		5.3	10.3		14 (5.5)	9 (15.5)	
Income, %			<0.001 (0.274)			<0.001			0.011
Low	183,080 (47.0)	6,863 (60.5)		15.2	24.8		75 (29.5)	28 (48.3)	
Middle	184,575 (47.4)	4,121 (36.3)		41.3	46.5		114 (44.9)	23 (39.7)	
High	21,695 (5.6)	361 (3.2)		43.5	28.7		65 (25.6)	7 (12.1)	
Healthy diet score, %			<0.001 (0.038)			0.002			0.013
Low adherence	18,698 (4.8)	680 (6.0)		22.5	28.4		61 (24.0)	24 (41.4)	
Moderate adherence	234,394 (60.2)	6,900 (60.8)		58.2	59.1		138 (54.3)	28 (48.3)	
High adherence	136,258 (35.0)	3,765 (33.2)		19.3	12.5		55 (21.7)	6 (10.3)	
Residence, %			0.715 (0.006)			-			0.098
Urban	335,246 (86.1)	9,795 (86.3)		-	-		181 (71.3)	33 (56.9)	
Rural	54,098 (13.9)	1,550 (13.7)		-	-		71 (28.0)	24 (41.4)	
Unknown	6 (0.0)	0 (0.0)		-	-		2 (0.8)	1 (1.7)	
PhenoAge acceleration, years	-1.73 (4.28)	-0.81 (5.03)	<0.001 (0.197)	−0.12 (0.06)	1.34 (0.21)	<0.001	−0.59 (4.10)	1.08 (4.95)	0.019
PhenoAge, years	54.30 (9.04)	59.31 (8.84)	<0.001 (0.558)	45.70 (0.36)	67.48 (0.75)	<0.001	55.72 (13.40)	67.08 (12.55)	<0.001
Biologically older, %	86,726 (22.3)	3,301 (29.1)	<0.001 (0.155)	41.5	54.2	<0.001	91 (35.8)	34 (58.6)	0.001

For the UK Biobank and the Second Xiangya Hospital, continuous variables are presented as mean (standard deviation) and categorical variables as n (%); the NHANES entries are survey-weighted and are presented as described below. BMI, body mass index; PIR, poverty-income ratio; PhenoAge, phenotypic age. BMI categories were defined as underweight, BMI <18.5 kg/m^2^; normal weight, 18.5 ≤ BMI<25 kg/m^2^; overweight, 25 ≤ BMI<30 kg/m^2^; and obesity, BMI ≥30 kg/m^2^. Healthy-diet adherence was categorized as low, moderate, and high adherence according to cohort-specific diet-quality scores. In the UK Biobank, healthy-diet adherence was based on the Healthy Diet Score; in NHANES, it was based on the Healthy Eating Index-2020. In the UK Biobank, stroke referred to incident stroke identified through linked health records; in NHANES, stroke referred to self-reported physician-diagnosed stroke. In the Second Xiangya Hospital, stroke referred to physician-diagnosed stroke status at the index clinical encounter. NHANES estimates are survey-weighted and account for the strata and primary sampling units of the complex multistage design; design-based Wald tests and Rao–Scott chi-square tests were used for the corresponding p values. UK Biobank and Second Xiangya Hospital estimates are unweighted. Standardised mean differences are reported in addition to p values for the UK Biobank because, at this sample size, differences of negligible magnitude reach conventional statistical significance.

To quantify how far the estimated association between biological ageing and stroke moved after diet quality was added to the adjustment set, exploratory change-in-estimate analyses were conducted with PhenoAge acceleration as the exposure, healthy-diet adherence as the diet-quality indicator, and stroke as the outcome. Healthy-diet adherence was modelled as a three-level variable: low, moderate, and high adherence. For the UK Biobank cohort, analyses were based on Cox proportional hazards models for incident stroke. For NHANES, analyses were based on survey-weighted logistic regression models for self-reported physician-diagnosed stroke status. For the Second Xiangya Hospital dataset, analyses were based on multivariable logistic regression models for physician-diagnosed stroke status at the index clinical encounter. Two sequential models were fitted. The base model estimated the association between PhenoAge acceleration and stroke after adjustment for covariates but without healthy-diet adherence. The diet-adjusted model additionally included healthy-diet adherence. The degree of attenuation associated with adjustment for healthy-diet adherence was calculated as the difference between the regression coefficients for PhenoAge acceleration in the base and diet-adjusted models on the log-risk or log-odds scale. The percentage attenuation was calculated using the following formula:

$$\frac{\beta_{\text{base}}-\beta_{\text{diet}-\text{adjusted}}}{\beta_{\text{base}}}\times 100\%$$

where $\beta_{\text{base}}$ represents the regression coefficient for PhenoAge acceleration before adjustment for healthy-diet adherence, and $\beta_{\text{diet}-\text{adjusted}}$ represents the corresponding coefficient after additional adjustment for healthy-diet adherence. Confidence intervals for the attenuation estimates were obtained using 8,000 bootstrap resampling iterations.

Because diet quality and PhenoAge acceleration were measured at the same assessment in NHANES and the Second Xiangya Hospital dataset, and because both datasets had cross-sectional designs, these analyses were interpreted as descriptive covariate-adjustment analyses rather than as evidence of causal natural direct or indirect effects. Two further points constrain the interpretation of this comparison. First, because the odds ratio and the hazard ratio are non-collapsible, part of any change in the coefficient across nested models reflects a change in the conditioning set and the associated rescaling rather than an explanatory contribution of the added covariate (29, 30); the absolute change in the regression coefficient is therefore reported alongside the percentage change, and the average-marginal-effect scale is additionally used in the hospital dataset (29). The component associations of diet quality with PhenoAge acceleration and with stroke are also reported. Exploratory subgroup analyses were conducted according to prespecified variables, including age, sex, educational level, BMI category, comorbidity index, smoking status, alcohol consumption, income or poverty-income ratio, sleep-duration group, healthy-diet adherence, and other dataset-specific variables where available. Race subgroup analyses were performed only in the UK Biobank and NHANES. Deprivation or poverty-income-ratio subgroup analyses were performed only in the UK Biobank and NHANES. Residence subgroup analyses were performed in the UK Biobank and the Second Xiangya Hospital dataset. These analyses were performed to examine whether the association between biological ageing status and stroke differed across population subgroups. Interaction terms between biological ageing status and each subgroup variable were tested in the fully adjusted models. *p* values for interaction were reported, and subgroup findings were interpreted cautiously because of multiple comparisons.

Sensitivity analyses were conducted in the UK Biobank cohort by excluding participants who developed stroke within the first three years of follow-up to reduce the possibility of reverse causation. Additional sensitivity analyses were performed by modelling healthy-diet adherence as an ordinal variable and by using continuous BMI instead of BMI category. For NHANES and the Second Xiangya Hospital dataset, sensitivity analyses were performed using alternative model specifications, including healthy-diet adherence as an ordinal variable and continuous BMI instead of BMI category. Detailed specifications and results are provided in Supplementary Tables S4–S10. Because only 58 stroke cases were available in the Second Xiangya Hospital dataset, the fully adjusted model estimated a large number of parameters relative to the number of events, with a correspondingly high risk of overfitting, unstable coefficients, quasi-complete separation and sparse-data bias (31, 32). We therefore prespecified a parsimonious core adjustment set for this dataset, comprising chronological age, sex, body mass index, comorbidity burden and smoking status, chosen on causal grounds rather than by statistical screening, and we report the total number of estimated parameters together with the number of events per parameter. All logistic-regression specifications converged. Firth’s penalised logistic regression was fitted as a sensitivity analysis to reduce small-sample bias and to protect against separation (33), and the stability of coefficients was assessed across 2,000 bootstrap resamples. The subgroup analyses in the hospital dataset are presented in the Supplementary material. Across all three datasets, subgroup findings are interpreted according to the formal test of interaction rather than the presence or absence of statistical significance within individual strata, since a significant association in one stratum and a non-significant association in another does not by itself establish effect modification; given the number of comparisons, interaction *p* values were additionally corrected using the Benjamini–Hochberg false-discovery-rate procedure (34), and the number of participants and stroke events in each stratum is reported. Missing data were handled by complete-case analysis in the primary models. This approach was retained as primary because it provides a transparent analysis of the observed data without imposing a model on the missing values. Multiple imputation and inverse-probability-of-completeness weighting were used to assess sensitivity to the handling of missing data. Variable-specific missing-data proportions for the PhenoAge biomarkers, the dietary variables and every covariate are reported for all three datasets in Supplementary Table S11, and included participants are compared with excluded participants in Supplementary Table S12. Because complete-case analysis can introduce selection bias when completeness is itself related to health status, socioeconomic position or survey cycle, the primary models were additionally refitted after multiple imputation by chained equations, with 20 imputed datasets and estimates pooled using Rubin’s rules, and after inverse-probability-of-completeness weighting (35).

All statistical analyses were performed using R software, version 4.3.2, with the survival package for the UK Biobank time-to-event analyses, the survey package for all design-based NHANES analyses, the mice package for multiple imputation, the cmprsk package for competing-risk models, and the logistf and rms packages for the penalised and spline analyses in the hospital dataset. A two-sided *p* value less than 0.05 was considered statistically significant. Package versions were survival 3.5.8, survey 4.4.2, mice 3.16.0, cmprsk 2.2.11, logistf 1.26.0, rms 6.8.0 and car 3.1.2.

## 3 Results

### 3.1 Characteristics of included participants

Table 1 presents the characteristics of included participants according to stroke status in the UK Biobank, NHANES, and the Second Xiangya Hospital. After excluding participants with missing data on exposure, outcome, diet quality, and covariates, a total of 400,695 participants from the UK Biobank, 21,754 participants from NHANES, and 312 participants from the Second Xiangya Hospital were included in the analysis. In the UK Biobank cohort, 11,345 participants developed stroke during a median follow-up of 15.83 years, corresponding to a cumulative stroke incidence of 2.8%. In NHANES, 729 participants reported a history of stroke, corresponding to 3.4% of the unweighted analytic sample. In the Second Xiangya Hospital cross-sectional dataset, 58 participants had physician-diagnosed stroke at the index clinical encounter, corresponding to a stroke proportion of 18.6%.

In the UK Biobank, compared with participants without stroke, those who developed stroke were older, more likely to be male, socioeconomically deprived, have lower educational attainment, higher BMI, greater comorbidity burden, and a higher prevalence of ever smoking. Participants who developed stroke were also more likely to report never or previous alcohol consumption, have short or long sleep duration, and belong to the low-income group. The proportion of obesity was higher among participants with stroke than among those without stroke. Healthy-diet adherence also differed by stroke status, with participants who developed stroke having a slightly higher proportion of low healthy-diet adherence and a lower proportion of high healthy-diet adherence. In contrast, residence distribution was similar between participants with and without stroke in the UK Biobank.

Similar patterns were observed in NHANES. Participants with stroke were substantially older than those without stroke and were more likely to have a low poverty-income ratio, lower educational attainment, higher BMI, greater comorbidity burden, and a higher prevalence of ever smoking. They were also more likely to report previous alcohol consumption, short or long sleep duration, and lower socioeconomic status based on the poverty-income ratio. Unlike the UK Biobank, the proportion of males did not differ significantly by stroke status in NHANES. Healthy-diet adherence differed between participants with and without stroke, with participants reporting stroke having a higher proportion of low adherence and a lower proportion of high adherence.

In the Second Xiangya Hospital dataset, participants with stroke were older than those without stroke and had lower educational attainment, greater comorbidity burden, and a higher prevalence of ever smoking. Alcohol consumption pattern also differed by stroke status, with participants with stroke more likely to report never or previous alcohol consumption and less likely to report current alcohol consumption. Sleep-duration group differed between participants with and without stroke, mainly because the stroke group had a higher proportion of long sleep duration. Participants with stroke were also more likely to belong to the low-income group and less likely to belong to the high-income group. Healthy-diet adherence showed a similar pattern to the population-based datasets, with a higher proportion of low adherence and a lower proportion of high adherence among participants with stroke. In contrast, sex, continuous BMI, BMI category, and residence distribution did not differ significantly by stroke status in the Second Xiangya Hospital dataset. Race and deprivation or poverty-income-ratio variables were not evaluated in the Second Xiangya Hospital dataset.

Across the three studies, biological ageing measures differed by stroke status. In the UK Biobank, participants who developed stroke had higher PhenoAge acceleration than those without stroke (−0.81 vs. −1.73 years), higher PhenoAge (59.31 vs. 54.30 years), and a higher proportion of biologically older individuals (29.1% vs. 22.3%). In NHANES, where all estimates are survey-weighted, participants with stroke also showed higher PhenoAge acceleration (1.34 vs. -0.12 years), higher PhenoAge (67.48 vs. 45.70 years), and a higher proportion of biologically older individuals (54.2% vs. 41.5%). In the Second Xiangya Hospital dataset, participants with stroke similarly had higher PhenoAge acceleration than those without stroke (1.08 vs. -0.59 years), higher PhenoAge (67.08 vs. 55.72 years), and a higher proportion of biologically older individuals (58.6% vs. 35.8%). Variable-specific missingness before exclusion in the UK Biobank was 18.4% for the PhenoAge biomarker panel, 15.3% for household income, 2.5% for the comorbidity index, 2.0% for educational level, 1.9% for body mass index, 1.7% for diet quality, and 1.1% or less for every remaining covariate (Supplementary Table S11). Included and excluded UK Biobank participants were closely comparable on all measured characteristics, with every standardised mean difference below 0.11, and the proportion with any recorded stroke diagnosis was almost identical in the two groups (2.83% versus 2.88%), indicating that complete-case selection is unlikely to have introduced material selection bias (Supplementary Table S12). Corresponding comparisons among eligible adults in NHANES and among all individuals screened for the hospital dataset are given in Supplementary Tables S12b,c; all standardised mean differences were below 0.15, with excluded NHANES participants slightly younger and of slightly lower poverty-income ratio. In the Second Xiangya Hospital dataset, variable-specific missingness before exclusion, calculated among the 391 individuals screened, was 4.9% for the PhenoAge biomarker panel, 3.8% for diet quality, 1.5% for educational level and 0.8% for residence.

### 3.2 Association between biological ageing and stroke

**Figure 2:**
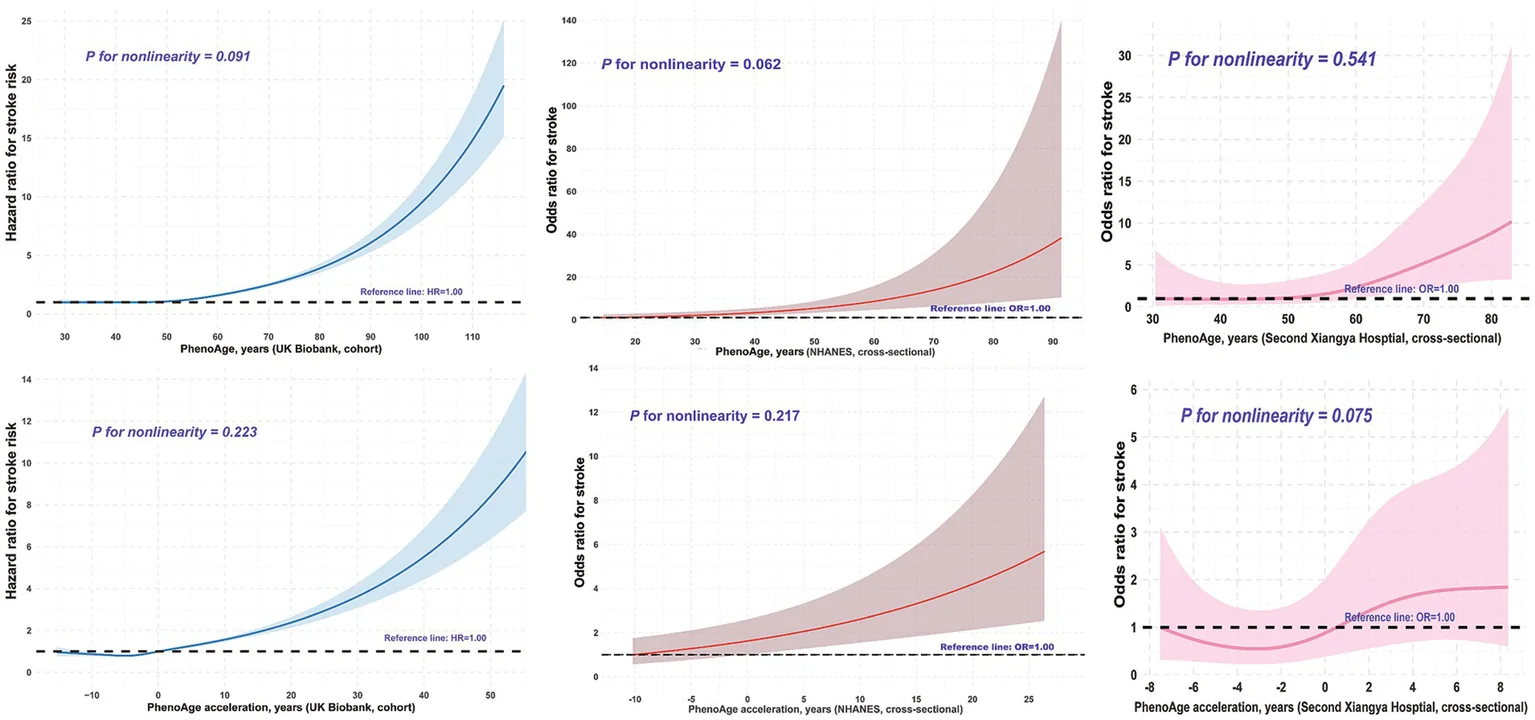
Restricted cubic spline analyses of the associations of PhenoAge and PhenoAge acceleration with stroke in the UK Biobank, NHANES, and the Second Xiangya Hospital. Restricted cubic spline models were used to examine the dose–response associations of PhenoAge and PhenoAge acceleration with stroke. The upper panels show the associations for PhenoAge, and the lower panels show the corresponding associations for PhenoAge acceleration. From left to right, the columns represent the UK Biobank cohort, NHANES cross-sectional study, and Second Xiangya Hospital cross-sectional dataset. Effect estimates are presented as HRs in the UK Biobank and ORs in NHANES and the Second Xiangya Hospital, with shaded areas indicating 95% confidence intervals. The dashed horizontal line represents the reference value (HR/OR = 1.00). *p* values for non-linearity were greater than 0.05 across all panels, supporting approximately linear associations between biological ageing measures and stroke in the three datasets. PhenoAge, phenotypic age. The four knots were placed at the 5th, 35th, 65th and 95th percentiles of the exposure distribution within each dataset, and non-linearity was assessed by a likelihood ratio test of the non-linear spline terms.

For the association between biological ageing and stroke risk or odds, the UK Biobank cohort analysis showed that accelerated biological ageing was significantly associated with a higher risk of incident stroke. Each 1-year increase in PhenoAge acceleration was associated with a 6.0% higher risk of stroke (HR = 1.060, 95% CI 1.056–1.064), and biologically older participants had a substantially higher risk of stroke than biologically younger participants (HR = 1.724, 95% CI 1.660–1.791) (Table 2). When PhenoAge acceleration was analysed by quartiles, a clear dose–response association was observed: compared with participants in the lowest quartile, the HRs for stroke were 1.382 (95% CI 1.288–1.483) for quartile 2, 1.861 (95% CI 1.731–2.001) for quartile 3, and 2.784 (95% CI 2.584–2.999) for quartile 4 (all *p*-values <0.001) (Table 2). Consistent findings were observed in the NHANES cross-sectional analysis. Each 1-year increase in PhenoAge acceleration was associated with a 5.8% higher odds of stroke (OR = 1.058, 95% CI 1.037–1.080), and biologically older participants had higher odds of stroke than biologically younger participants (OR = 1.683, 95% CI 1.329–2.132) (Table 2). In the quartile analysis, compared with quartile 1, the ORs for stroke were 1.421 (95% CI 0.981–2.058) for quartile 2, 2.046 (95% CI 1.450–2.886) for quartile 3, and 3.482 (95% CI 2.541–4.772) for quartile 4. Although the association for quartile 2 did not reach statistical significance (*p* = 0.064), higher quartiles of PhenoAge acceleration were significantly associated with increased odds of stroke. In the Second Xiangya Hospital cross-sectional analysis, accelerated biological ageing was also associated with higher odds of physician-diagnosed stroke at the index clinical encounter. Each 1-year increase in PhenoAge acceleration was associated with an 8.2% higher odds of stroke (OR = 1.082, 95% CI 1.002–1.169; *p* = 0.045), and biologically older participants had higher odds of stroke than biologically younger participants (OR = 1.786, 95% CI 1.102–2.895; *p* = 0.019) (Table 2). In the quartile analysis, compared with quartile 1, the ORs for stroke were 1.214 (95% CI 0.778–1.893; *p* = 0.392) for quartile 2, 1.672 (95% CI 1.014–2.758; *p* = 0.044) for quartile 3, and 1.934 (95% CI 1.130–3.310; *p* = 0.016) for quartile 4. Although quartile 2 was not statistically significant, quartiles 3 and 4 showed significantly higher odds of stroke, supporting a broadly dose-dependent association between higher PhenoAge acceleration and stroke odds in the hospital-based cross-sectional dataset. Restricted cubic spline analyses further supported an approximately linear exposure-response relationship between PhenoAge acceleration and stroke in all three datasets, with *p*-values for non-linearity greater than 0.05 (Figure 2). Estimates expressed per one standard deviation of PhenoAge acceleration, per five-year increase in the UK Biobank, and after redefining PhenoAge acceleration as the residual of PhenoAge regressed on chronological age, together with the correlation between PhenoAge acceleration and chronological age and the variance inflation factors for the primary models, are presented in Supplementary Table S13; the sequential Models 1 to 4 and the models excluding potentially downstream comorbidities are shown in Supplementary Table S14; and the formal test of the proportional-hazards assumption, incidence rates per 1,000 person-years, competing-risk estimates, subtype-specific analyses for ischaemic and haemorrhagic stroke, the five-year and seven-year lag analyses, and a sensitivity analysis censoring the whole cohort at the earliest national linkage end date are shown in Supplementary Table S15. Estimates after multiple imputation, inverse-probability-of-completeness weighting, penalised logistic regression, use of the parsimonious hospital adjustment set, and analyses on the average-marginal-effect scale are shown in Supplementary Table S16. Across these specifications the direction of the association was stable, although its statistical significance varied with the model chosen in the smaller hospital dataset. Two features of the UK Biobank analysis deserve emphasis. First, the association differed markedly by stroke subtype: PhenoAge acceleration was associated with ischaemic stroke (7,459 events; HR 1.066, 95% CI 1.061–1.071) but showed no clear association with haemorrhagic stroke (3,886 events; HR 1.009, 95% CI 0.998–1.020). Second, formal testing indicated that the proportional-hazards assumption was not satisfied for the exposure term (χ^2^ = 13.83, df = 1, *p* = 2.0 × 10^−4^; global χ^2^ = 209.63, df = 27, *p* < 0.001), and period-specific estimates showed a hazard ratio that declined across follow-up, from 1.071 (95% CI 1.062–1.080) within the first five years to 1.063 (95% CI 1.056–1.070) between five and ten years and 1.051 (95% CI 1.046–1.056) thereafter; the reported overall hazard ratio should therefore be read as an average effect over follow-up. Incidence rates were 2.00 and 3.01 per 1,000 person-years in biologically younger and biologically older participants respectively, and accounting for death as a competing event gave a subdistribution hazard ratio of 1.041 (95% CI 1.037–1.045). In the UK Biobank the Pearson correlation between chronological age and PhenoAge was 0.878, whereas the correlation between chronological age and PhenoAge acceleration was −0.021; the corresponding values in the Second Xiangya Hospital dataset were 0.616 and 0.108. Variance inflation factors for every term in the primary models did not exceed 1.11 in the UK Biobank or 1.95 in the Second Xiangya Hospital dataset, and the variance inflation factor for PhenoAge acceleration itself was 1.07 and 1.05 respectively, indicating that simultaneous adjustment for chronological age did not induce material collinearity. The standard deviation of PhenoAge acceleration was 4.36 years in the UK Biobank and 4.31 years in the Second Xiangya Hospital dataset.

**Table 2:** Association between biological ageing and the risk/odds of stroke.

Variables	UK Biobank cohort		NHANES cross-sectional		Second Xiangya Hospital cross-sectional	
	HR (95% CI)	*P*-value	OR (95% CI)	*P*-value	OR (95% CI)	*P*-value
Biological ageing status						
Biologically younger	1.000 (Reference)		1.000 (Reference)		1.000 (Reference)	
Biologically older	1.724 (1.660–1.791)	<0.001	1.683 (1.329–2.132)	<0.001	1.786 (1.102–2.895)	0.019
PhenoAge acceleration						
Quartile 1	1.000 (Reference)		1.000 (Reference)		1.000 (Reference)	
Quartile 2	1.382 (1.288–1.483)	<0.001	1.421 (0.981–2.058)	0.064	1.214 (0.778–1.893)	0.392
Quartile 3	1.861 (1.731–2.001)	<0.001	2.046 (1.450–2.886)	<0.001	1.672 (1.014–2.758)	0.044
Quartile 4	2.784 (2.584–2.999)	<0.001	3.482 (2.541–4.772)	<0.001	1.934 (1.130–3.310)	0.016
Continuous, per 1-year increase	1.060 (1.056–1.064)	<0.001	1.058 (1.037–1.080)	<0.001	1.082 (1.002–1.169)	0.045

In the UK Biobank cohort study, HRs and 95%CIs for incident stroke were estimated using Cox proportional hazards regression models. In the NHANES cross-sectional study, ORs and 95% CIs for self-reported physician-diagnosed stroke status were estimated using survey-weighted multivariable logistic regression models. In the Second Xiangya Hospital cross-sectional study, ORs and 95% CIs for physician-diagnosed stroke status at the index clinical encounter were estimated using multivariable logistic regression models. PhenoAge acceleration was analysed per 1-year increase, as biologically older versus younger, and by quartiles, with the biologically younger group and the lowest quartile as references. Models were adjusted for dataset-specific covariates; race and deprivation or poverty-income ratio were not evaluated in the Second Xiangya Hospital dataset. Diet quality was not included in this base model. PhenoAge, phenotypic age; BMI, body mass index; TDI, Townsend Deprivation Index; PIR, poverty-income ratio.

### 3.3 Subgroup analyses for the associations between biological ageing and stroke

**Figure 3:**
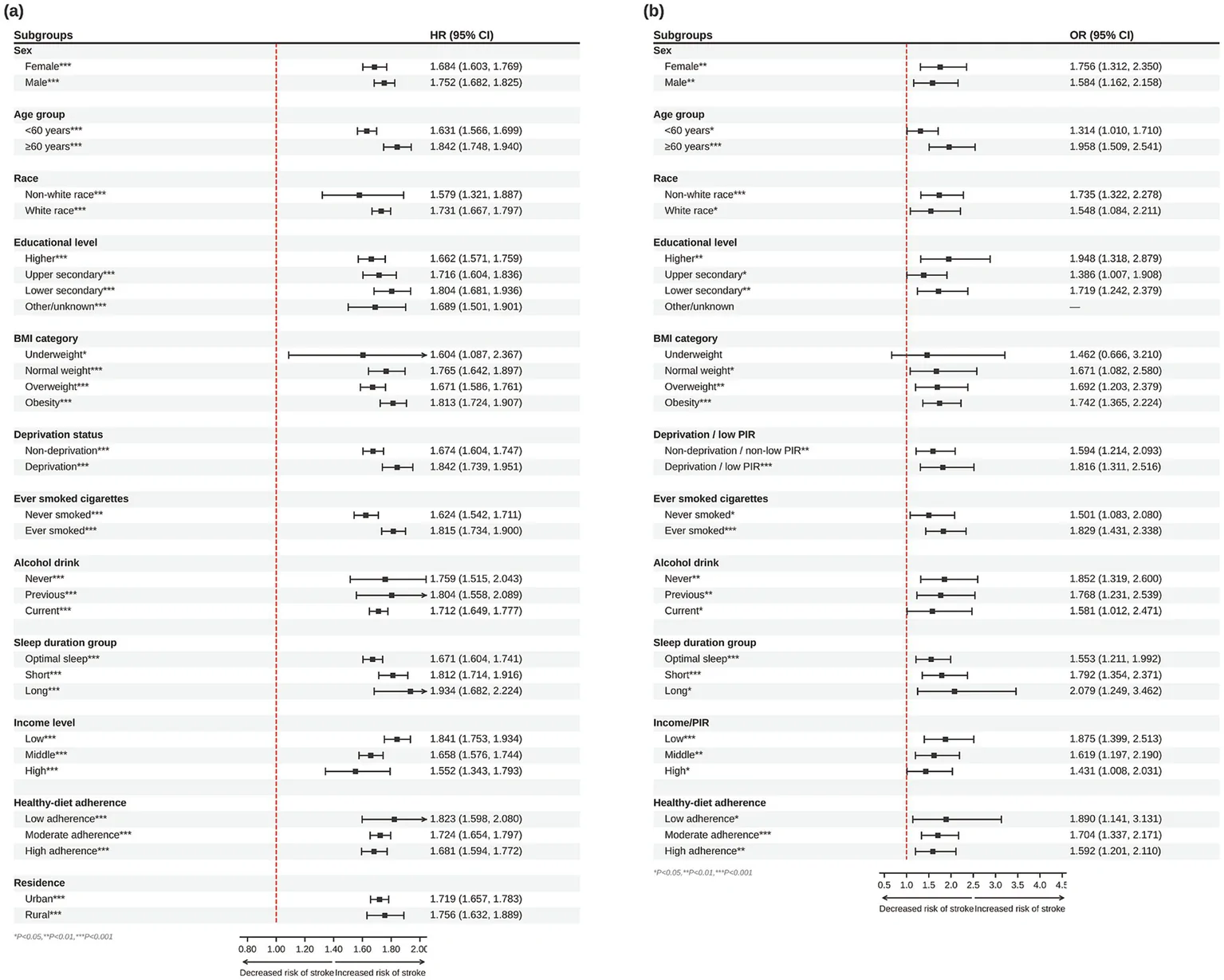
Exploratory subgroup analyses of the associations between biological ageing status and stroke in the UK Biobank and NHANES. Exploratory subgroup analyses were performed across prespecified population subgroups. **(a)** Shows the subgroup analyses in the UK Biobank cohort, with effect estimates presented as HRs. **(b)** Shows the subgroup analyses in NHANES, with effect estimates presented as ORs. Effect estimates compare biologically older participants with biologically younger participants within each subgroup. Models were adjusted for the same covariates as the primary models, except for the corresponding stratification variable where appropriate. *p* values for interaction were estimated by adding a product term between biological ageing status and the subgroup variable to the fully adjusted model. Horizontal lines indicate 95% confidence intervals, and the vertical dashed line represents the reference value of HR/OR = 1.00. *p < 0.05; ** *p* < 0.01; ***p < 0.001. Interaction *p* values were corrected using the Benjamini–Hochberg procedure. Hospital dataset analyses were based on 58 stroke cases and are correspondingly imprecise.

Exploratory subgroup analyses were conducted across prespecified population subgroups, and interaction terms between biological ageing status and each subgroup variable were tested in the fully adjusted models (Figure 3, showing the UK Biobank and NHANES). Overall, the association between being biologically older and stroke was observed across most subgroups in both the UK Biobank and NHANES, although the magnitude of association varied across strata. All subgroup analyses are exploratory, and interaction *p* values were corrected using the Benjamini–Hochberg procedure (Supplementary Table S17).

In the UK Biobank cohort, the association between being biologically older and incident stroke appeared more pronounced among participants aged ≥60 years (HR 1.842, 95% CI 1.748–1.940), those with socioeconomic deprivation (HR 1.842, 95% CI 1.739–1.951), ever smokers (HR 1.815, 95% CI 1.734–1.900), participants with long sleep duration (HR 1.934, 95% CI 1.682–2.224), those with low income (HR 1.841, 95% CI 1.753–1.934), and those with low healthy-diet adherence (HR 1.823, 95% CI 1.598–2.080). Relatively weaker associations were observed among participants aged <60 years, non-White participants, underweight individuals, non-deprived participants, never smokers, those with high income, and participants with high healthy-diet adherence.

In NHANES, broadly similar patterns were observed. Being biologically older was associated with higher odds of self-reported physician-diagnosed stroke in most subgroups. The association appeared more pronounced among participants aged ≥60 years (OR 1.958, 95% CI 1.509–2.541), those with deprivation or low PIR (OR 1.816, 95% CI 1.311–2.516), ever smokers (OR 1.829, 95% CI 1.431–2.338), participants with long sleep duration (OR 2.079, 95% CI 1.249–3.462), those with low income/PIR (OR 1.875, 95% CI 1.399–2.513), and those with low healthy-diet adherence (OR 1.890, 95% CI 1.141–3.131). The association was comparatively weaker among participants aged <60 years, underweight individuals, never smokers, those with high income/PIR, and participants with high healthy-diet adherence.

In the Second Xiangya Hospital cross-sectional dataset, which contained only 58 stroke cases in total, the stratified estimates were directionally consistent with those from the UK Biobank and NHANES but were derived from very small strata. These analyses have therefore been relocated to Supplementary Table S18 and are summarised only briefly here. Stratum-specific estimates were uniformly imprecise, and the full set is tabulated there rather than in the main text. Race and deprivation or poverty-income-ratio subgroups were not evaluated in the Second Xiangya Hospital dataset. None of the interaction tests in this dataset remained statistically significant after false-discovery-rate correction, and these stratified estimates should therefore be regarded as exploratory observations rather than as evidence of effect modification. Full results are in Supplementary Table S18.

### 3.4 Attenuation of the association between biological ageing and stroke after adjustment for diet quality

**Figure 4:**
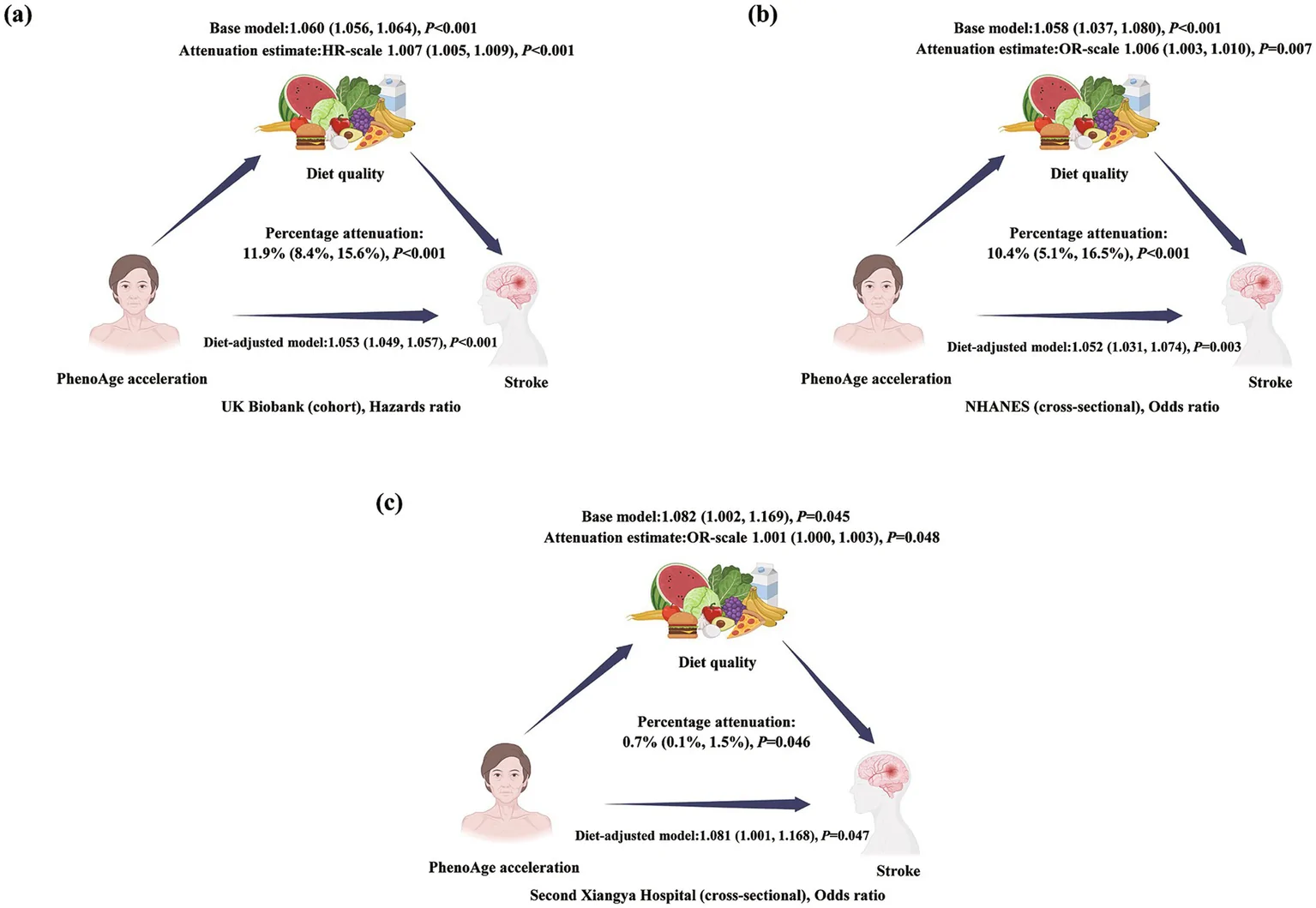
Attenuation of the association between PhenoAge acceleration and stroke after adjustment for diet quality. **(a)** Shows the UK Biobank cohort analysis, with incident stroke as the outcome and estimates presented as HRs. **(b,c)** Show the NHANES and Second Xiangya Hospital cross-sectional analyses, respectively, with stroke status as the outcome and estimates presented as ORs. PhenoAge acceleration was modelled per 1-year increase, and diet quality was assessed using cohort-specific healthy-diet adherence categories. The base model excluded healthy-diet adherence, whereas the diet-adjusted model additionally included it. The change in estimate was calculated from the difference in regression coefficients between the two models on the log-risk or log-odds scale, and is reported both as an absolute difference and as a percentage. Models were adjusted for dataset-specific covariates, including demographic, lifestyle, clinical, and available socioeconomic factors.

Adjustment for healthy-diet adherence modestly attenuated the association between PhenoAge acceleration and stroke in the UK Biobank, NHANES, and the Second Xiangya Hospital (Figure 4). In the UK Biobank cohort, each 1-year increase in PhenoAge acceleration was associated with a higher risk of incident stroke in the base model without healthy-diet adherence (HR 1.060, 95% CI 1.056–1.064; *p* < 0.001). After additional adjustment for healthy-diet adherence, the association remained statistically significant and was slightly attenuated (HR 1.053, 95% CI 1.049–1.057; *p* < 0.001). The attenuation associated with healthy-diet adherence corresponded to an estimate of 1.007 on the hazard ratio scale (95% CI 1.005–1.009; *p* < 0.001), representing an 11.9% attenuation of the association between PhenoAge acceleration and incident stroke (95% CI 8.4–15.6%; *p* < 0.001). These findings indicate that additional adjustment for healthy-diet adherence produced only a small change in the estimated association between accelerated biological ageing and incident stroke, and that the association was largely unchanged after diet quality was added to the model. Consistent findings were observed in the NHANES cross-sectional analysis. Each 1-year increase in PhenoAge acceleration was associated with higher odds of self-reported physician-diagnosed stroke in the base model without healthy-diet adherence (OR 1.058, 95% CI 1.037–1.080; *p* < 0.001). After additional adjustment for healthy-diet adherence, the association remained statistically significant and was modestly attenuated (OR 1.052, 95% CI 1.031–1.074; *p* = 0.003). The attenuation associated with healthy-diet adherence corresponded to an estimate of 1.006 on the odds ratio scale (95% CI 1.003–1.010; *p* = 0.007), representing a 10.4% attenuation of the association (95% CI 5.1–16.5%; *p* < 0.001). Similar but slightly weaker attenuation was observed in the Second Xiangya Hospital cross-sectional analysis. In the base model, each 1-year increase in PhenoAge acceleration was associated with higher odds of physician-diagnosed stroke at the index clinical encounter (OR 1.082, 95% CI 1.002–1.169; *p* = 0.045). After additional adjustment for healthy-diet adherence, the association remained statistically significant but was modestly attenuated (OR 1.081, 95% CI 1.001–1.168; *p* = 0.047). The attenuation associated with healthy-diet adherence corresponded to an estimate of 1.001 on the odds ratio scale (95% CI 1.000–1.003; *p* = 0.048), representing a 0.7% attenuation of the association (95% CI 0.1–1.5%; *p* = 0.046). Consequently, additional adjustment for diet quality produced a small but statistically detectable reduction in the coefficient for PhenoAge acceleration in all three datasets. Expressed on the scale on which it was calculated, the absolute change in the regression coefficient was 0.0070 (95% CI 0.0050–0.0090) in the UK Biobank, 0.0060 (95% CI 0.0030–0.0100) in NHANES and 0.0006 (95% CI 0.0001–0.0015) in the Second Xiangya Hospital, corresponding to shifts in the effect estimate from HR 1.060 to HR 1.053, from OR 1.058 to OR 1.052, and from OR 1.082 to OR 1.081, respectively. Part of this change may reflect the non-collapsibility of nested models rather than an explanatory contribution of diet quality. The component associations required to interpret this comparison were as follows. In the UK Biobank, better diet quality was associated with lower PhenoAge acceleration in the direction assumed by the causal framework (moderate versus low adherence, −0.413 years, 95% CI -0.476 to −0.350; high versus low adherence, −0.746 years, 95% CI − 0.811 to −0.681; both *p* < 0.001), whereas its association with incident stroke was weak and not statistically significant after full adjustment (moderate versus low adherence, HR 0.985, 95% CI 0.931–1.042; high versus low adherence, HR 0.962, 95% CI 0.908–1.019). In NHANES, higher diet quality measured by the Healthy Eating Index-2020 was similarly associated with lower PhenoAge acceleration (moderate versus low adherence, −0.382 years, 95% CI -0.455 to −0.309; high versus low adherence, −0.695 years, 95% CI -0.781 to −0.609; both *p* < 0.001), while its direct association with stroke was not statistically significant in the fully adjusted model (moderate versus low adherence, OR 0.951, 95% CI 0.835–1.082; high versus low adherence, OR 0.914, 95% CI 0.785–1.065). In the Second Xiangya Hospital dataset neither component association reached statistical significance (diet quality and stroke: moderate versus low adherence, OR 0.885, 95% CI 0.435–1.802; high versus low adherence, OR 0.762, 95% CI 0.285–2.038), which is consistent with the very small number of events and further limits what the change-in-estimate comparison can support in that dataset. All component associations between diet quality and stroke are tabulated in Supplementary Table S19. On the collapsible average-marginal-effect scale in the hospital dataset, additional adjustment for diet quality changed the absolute risk difference per 1-year increase in PhenoAge acceleration from 0.00957 to 0.00947, a change of 0.00009 corresponding to approximately 1.0% of the base-model effect.

### 3.5 Sensitivity analyses

Sensitivity analyses in the UK Biobank cohort yielded results broadly consistent with the primary analyses. After excluding participants who developed stroke within the first three years of follow-up, the association between PhenoAge acceleration and incident stroke remained significant. Each 1-year increase in PhenoAge acceleration was associated with a higher risk of incident stroke (HR 1.058, 95% CI 1.054–1.062; *p* < 0.001), and biologically older participants continued to show a higher stroke risk than biologically younger participants (HR 1.701, 95% CI 1.634–1.772; *p* < 0.001). The dose–response association across quartiles was also preserved, with HRs of 1.361 (95% CI 1.267–1.462), 1.828 (95% CI 1.698–1.968), and 2.704 (95% CI 2.503–2.921) for quartiles 2, 3, and 4, respectively, compared with quartile 1. The attenuation associated with healthy-diet adherence was also robust after excluding participants who developed stroke within the first three years of follow-up. The attenuation estimate on the hazard ratio scale was 1.006 (95% CI 1.004–1.009; *p* < 0.001), corresponding to an 11.2% attenuation of the association between PhenoAge acceleration and incident stroke after adjustment for healthy-diet adherence (95% CI 7.9–14.9%; *p* < 0.001). When healthy-diet adherence was modelled as an ordinal variable, the association in the base model without healthy-diet adherence remained significant (HR 1.060, 95% CI 1.056–1.064), and the association remained materially unchanged after additional adjustment for healthy-diet adherence (HR 1.054, 95% CI 1.050–1.058). The attenuation estimate associated with healthy-diet adherence was 1.006 on the hazard ratio scale (95% CI 1.004–1.008), corresponding to a 10.7% attenuation of the association (95% CI 7.5–14.3%). Similar findings were observed when BMI was modelled as a continuous variable instead of BMI category. The association in the base model without healthy-diet adherence was 1.059 (95% CI 1.055–1.063), and the association after additional adjustment for healthy-diet adherence was 1.052 (95% CI 1.048–1.056). The attenuation estimate associated with healthy-diet adherence was 1.007 on the hazard ratio scale (95% CI 1.005–1.009), corresponding to an 11.6% attenuation of the association (95% CI 8.2–15.2%). Because the Second Xiangya Hospital dataset had a cross-sectional design, exclusion of early incident stroke events was not applicable. Sensitivity analyses in this dataset therefore focused on alternative model specifications. When BMI was modelled as a continuous covariate, the association between PhenoAge acceleration and stroke remained statistically significant, although the estimates were slightly attenuated compared with the primary analysis. Each 1-year increase in PhenoAge acceleration was associated with higher odds of stroke (OR 1.049, 95% CI 1.009–1.091; *p* = 0.016), and biologically older participants had higher odds of stroke than biologically younger participants (OR 1.742, 95% CI 1.071–2.833; *p* = 0.026). Compared with quartile 1, the ORs were 1.198 (95% CI 0.762–1.883; *p* = 0.431) for quartile 2, 1.641 (95% CI 1.003–2.686; *p* = 0.049) for quartile 3, and 1.901 (95% CI 1.108–3.262; *p* = 0.020) for quartile 4. When healthy-diet adherence was modelled as an ordinal variable in the Second Xiangya Hospital dataset, the association remained significant in the base model (OR 1.078, 95% CI 1.003–1.158; *p* = 0.041) and after additional adjustment for healthy-diet adherence (OR 1.074, 95% CI 1.000–1.153; *p* = 0.049). The attenuation estimate was 1.004 on the odds ratio scale (95% CI 1.001–1.008; *p* = 0.047), corresponding to a 4.8% attenuation of the association (95% CI 0.2–10.3%; *p* = 0.046). When BMI was modelled as a continuous covariate in the change-in-estimate analysis, the base model estimate was 1.081 (95% CI 1.006–1.163; *p* = 0.034), and the diet-adjusted estimate was 1.076 (95% CI 1.002–1.156; *p* = 0.044). The attenuation estimate was 1.005 on the odds ratio scale (95% CI 1.001–1.009; *p* = 0.039), corresponding to a 5.7% attenuation of the association (95% CI 0.5–11.5%; *p* = 0.034). Overall, these findings indicate that the observed association between accelerated biological ageing and stroke was directionally stable across alternative analytic specifications, although its statistical significance in the small hospital dataset varied with the specification chosen. The small change after adjustment for healthy-diet adherence was similarly consistent across sensitivity analyses, supporting the interpretation that the coefficient for PhenoAge acceleration moved only slightly when diet quality was added to the adjustment set, without implying causal mediation. Detailed estimates for the exclusion of early stroke events, attenuation after early-event exclusion, ordinal healthy-diet adherence, continuous BMI, and Second Xiangya Hospital sensitivity analyses are shown in Supplementary Tables S4–S10. Corresponding sensitivity analyses using multiple imputation, inverse-probability-of-completeness weighting, competing-risk models, the parsimonious hospital adjustment set, Firth’s penalised logistic regression and bootstrap assessment of coefficient stability are reported in Supplementary Tables S15, S16. In the fully adjusted hospital model 18 parameters were estimated from 58 events, corresponding to approximately 3.2 events per estimated parameter. Under the parsimonious core adjustment set the estimate was essentially unchanged, and Firth penalised regression produced a very similar coefficient with slightly wider uncertainty; across 2,000 bootstrap resamples the coefficient was positive in 96.4% of replications, with a percentile interval that included the null. These results indicate that the direction of the hospital-based association is stable but that its statistical significance is fragile, and they are reported in full in Supplementary Table S16.

## 4 Discussion

In this study, we used data from a large prospective UK cohort, a nationally representative US cross-sectional study, and a hospital-based Chinese cross-sectional study to examine the association between biological ageing, diet quality, and stroke. In the UK Biobank cohort, accelerated biological ageing, measured by PhenoAge acceleration, was associated with a higher risk of incident stroke, and a clear dose–response pattern was observed across quartiles of PhenoAge acceleration. Directionally similar findings were observed in NHANES, where higher PhenoAge acceleration was associated with higher odds of prevalent self-reported physician-diagnosed stroke, and in the Second Xiangya Hospital, where higher PhenoAge acceleration was associated with higher odds of prevalent physician-documented stroke at the index clinical encounter. Because the NHANES and hospital analyses are cross-sectional, agreement in direction does not establish a temporally consistent relationship. Restricted cubic spline analyses supported an approximately linear exposure-response relationship across the three datasets. We further found that additional adjustment for healthy-diet adherence modestly attenuated the association between PhenoAge acceleration and stroke, with changes in the log-scale coefficient of 11.9% in the UK Biobank, 10.4% in NHANES and 0.7% in the Second Xiangya Hospital, corresponding to absolute changes of less than 0.01 on the log-hazard or log-odds scale in every dataset. Exploratory subgroup analyses across prespecified variables suggested that the association between biological ageing status and stroke may be more pronounced among participants with older chronological age, smoking history, long sleep duration, lower income, and poorer diet quality, as well as among socioeconomically disadvantaged participants in datasets where deprivation or poverty-income ratio was available. To our knowledge, this study is the first to quantify explicitly, and in parallel across a prospective cohort, a nationally representative survey and an exploratory clinical sample, how little of the estimated association between accelerated biological ageing and stroke is displaced when diet quality is added to the adjustment set. This comparison describes covariate adjustment rather than causal pathway decomposition, and causal mediation cannot be inferred.

Our findings are consistent with previous evidence linking biological ageing to stroke and related neurological outcomes. Zhang et al. analysed 253,932 UK Biobank participants and documented 5,460 incident strokes during a median follow-up of 13.6 years. They reported that each 1-SD increase in PhenoAge acceleration was associated with higher risk of total stroke (HR 1.22, 95% CI 1.19–1.25), ischaemic stroke (HR 1.26, 95% CI 1.22–1.29), intracerebral haemorrhage (HR 1.08, 95% CI 1.02–1.16), and subarachnoid haemorrhage (HR 1.08, 95% CI 1.00–1.18), concluding that accelerated biological ageing may identify individuals with elevated stroke susceptibility (8). Our subtype analysis is only partly concordant with that report: we observed a clear association with ischaemic stroke but no material association with haemorrhagic stroke, which may be compatible with a stronger relationship with ischaemic than with haemorrhagic stroke, without establishing the underlying pathway, and which indicates that estimates for total stroke are driven mainly by its ischaemic component (8). Mak et al. studied 325,870 UK Biobank participants without neurological disease at baseline and found that clinical biomarker-based biological ageing was associated with subsequent neurological disorders, including ischaemic stroke, supporting the value of blood chemistry-derived ageing measures for neurological risk stratification (7). Wang et al. further extended this evidence to stroke prognosis by analysing 7,396 patients with acute ischaemic stroke or transient ischaemic attack from a national registry in China. Compared with patients in the lowest PhenoAge acceleration group, those in the highest group had higher risks of recurrent stroke, ischaemic stroke, composite vascular events, all-cause death, and poor functional outcome at 3 months (36). In addition, Wang et al. used NHANES data and Mendelian randomization methods and reported that biological ageing indicators were associated with stroke and post-stroke mortality; genetically predicted frailty index was associated with overall stroke, while reverse Mendelian randomization suggested that stroke liability was associated with greater PhenoAge acceleration (37). Compared with these previous studies, our study adds novel evidence by quantifying explicitly how far the estimated association between accelerated biological ageing and stroke moves when diet quality is added to the adjustment set, and by extending the analysis from population-based datasets to an exploratory hospital-based Chinese cross-sectional setting.

The direction of the change in estimate associated with diet quality is biologically plausible. PhenoAge integrates chronological age with biomarkers reflecting inflammation, renal function, glucose metabolism, immune status, haematological ageing, and liver-related physiology. Poor diet quality may contribute to accelerated ageing through chronic low-grade inflammation, oxidative stress, insulin resistance, endothelial dysfunction, dyslipidaemia, and metabolic disturbance. These pathways may influence PhenoAge components such as glucose, C-reactive protein, albumin, white blood cell count, lymphocyte percentage, red cell distribution width, and alkaline phosphatase, while also increasing vascular vulnerability. Evidence from prior studies supports the link between dietary patterns and biological ageing. Kresovich et al. analysed 2,694 women and found that healthier eating patterns were associated with lower epigenetic age acceleration; each 1-SD increase in Alternative Healthy Eating Index-2010 was associated with lower PhenoAge acceleration and GrimAge acceleration, suggesting that better diet quality may reflect slower biological ageing (9). Wang et al. analysed 16,666 NHANES participants and found that healthy dietary patterns were associated with decelerated biological ageing, whereas a higher dietary inflammatory index was associated with greater odds of accelerated PhenoAge, with the highest dietary inflammatory index category associated with accelerated PhenoAge (OR 1.61, 95% CI 1.39–1.87) (10). Cardoso et al. examined 16,055 NHANES adults aged 20 to 79 years and reported that each 10% higher energy intake from ultra-processed foods was associated with being 0.21 years biologically older in terms of PhenoAge (95% CI 0.16–0.26), while adjustment for diet quality attenuated the association, suggesting that diet quality accounts for part, but not all, of the food processing-related ageing signal (38). The small magnitude of the change in the coefficient is unsurprising because PhenoAge captures multiple physiological systems, and stroke is a multifactorial outcome influenced by blood pressure, diabetes, atrial fibrillation, lipids, kidney function, inflammation, socioeconomic conditions, and lifestyle factors. Beyond conventional vascular risk factors, broader ageing-related vulnerability may also be shaped by environmental exposures, frailty-related processes, and chronic disease-related molecular alterations, including kidney-related biological pathways (39, 40). Because NHANES and the Second Xiangya Hospital dataset were cross-sectional, and diet was measured at one assessment point, these findings should be interpreted as a descriptive change-in-estimate comparison rather than as evidence of a causal pathway. In the cross-sectional datasets, reverse causation cannot be excluded: stroke and its management alter PhenoAge biomarkers and dietary habits (12). The lag analysis addresses this concern only in the prospective UK Biobank.

Exploratory subgroup analyses were conducted across prespecified population subgroups. In the UK Biobank and NHANES, the association between being biologically older and stroke appeared to be more pronounced among participants aged 60 years or older, ever smokers, participants with long sleep duration, those with lower income, and those with low healthy-diet adherence. In the Second Xiangya Hospital dataset the stratified estimates were directionally similar, but no interaction remained statistically significant after false-discovery-rate correction and every stratum contained very few events, so that dataset contributes no independent evidence of effect modification. Socioeconomic deprivation or low PIR showed similar patterns in the UK Biobank and NHANES, whereas these variables were not evaluated in the Second Xiangya Hospital dataset. These findings are consistent with the view that accelerated biological ageing may have stronger vascular consequences in individuals already exposed to social, behavioural, or physiological vulnerability. In the INTERSTROKE analysis of tobacco exposure across 32 countries, current smoking was associated with higher odds of all stroke and showed a stronger association with ischaemic stroke than with intracerebral haemorrhage, supporting smoking as a major vascular risk amplifier (41). Chen et al. analysed 409,156 adults from the China Kadoorie Biobank and reported a U-shaped association between sleep duration and stroke; compared with 7 to 8 h, sleep duration of 5 h or less was associated with higher total stroke risk, and sleep duration of 10 h or more was also associated with higher total stroke risk (42). Foster et al. analysed 328,594 UK Biobank participants and found significant interactions between lifestyle score and socioeconomic deprivation for all-cause and cardiovascular mortality; compared with healthier lifestyle, the less healthy lifestyle category was associated with higher all-cause mortality in the least deprived quintile, while the corresponding risk was greater in the most deprived quintile according to the reported study findings (43). Related evidence also suggests that behavioural factors may interact with environmental exposures in shaping cardiometabolic risk. For example, a prospective cohort study in China reported that physical activity was involved in the relationship between multiple air-pollutant exposures and hypertension, a major vascular risk factor (44). These subgroup findings are exploratory and hypothesis-generating, interpreted according to the formal interaction tests after false-discovery-rate correction.

This study has several strengths. First, we used three complementary datasets, including a prospective UK cohort for incident stroke, a nationally representative US survey for stroke status, and a hospital-based Chinese cross-sectional dataset for physician-diagnosed stroke status in a clinical setting (45, 46). Second, PhenoAge was calculated using routinely available clinical biomarkers, which may improve translational relevance compared with more costly molecular ageing measures (46, 47). Third, we examined biological ageing in multiple ways, including continuous PhenoAge acceleration, binary biological ageing status, quartiles, and restricted cubic splines. Fourth, we assessed attenuation after adjustment for diet quality, exploratory subgroup differences, and sensitivity analyses, while incorporating survey design features in NHANES (26). Fifth, the inclusion of the Second Xiangya Hospital dataset provided a clinically oriented setting in which the association could be examined using routinely collected hospital information, although this dataset is exploratory and is not presented as validation. Several limitations should also be acknowledged. First, the observational design precludes causal inference, and residual confounding from unmeasured or imperfectly measured factors may remain (48, 49). Second, stroke in NHANES was based on self-reported physician diagnosis, and the cross-sectional design limits temporal interpretation; previous validation studies have shown that self-reported stroke can be useful in epidemiological settings but may be affected by imperfect sensitivity and specificity (50, 51). Similarly, the Second Xiangya Hospital dataset was cross-sectional, and although stroke status was based on physician diagnosis and clinical records, the temporal sequence between biological ageing, diet quality, and stroke could not be established. Third, PhenoAge biomarkers and diet quality were measured at baseline, survey assessment, or the index clinical encounter, so changes in biological ageing and dietary habits over time could not be captured (52, 53). Fourth, diet quality was assessed using cohort-specific indices, which improves internal validity within each study but may limit direct comparability across the UK Biobank, NHANES, and the Second Xiangya Hospital (54, 55). Fifth, the UK Biobank population was predominantly White and healthier than the general population, which may affect generalizability (56). The Second Xiangya Hospital sample was hospital-based and relatively small, which may introduce selection bias and limit precision in subgroup and sensitivity analyses. No formal external validation was undertaken in any dataset: no externally derived prediction equation was transported, and discrimination, calibration, reclassification and decision-curve analyses were not performed, so the hospital sample constitutes an exploratory clinical dataset rather than a validation dataset. Sixth, in the UK Biobank cohort, death before stroke was treated as a censoring event, so the primary Cox model estimates the cause-specific hazard rather than the effect on the cumulative incidence of stroke; a Fine–Gray subdistribution hazard model was fitted as a complementary analysis, but competing mortality may nonetheless influence how the primary estimate should be read. Seventh, the change-in-estimate analysis was based on differences in regression coefficients after adjustment for diet quality and should not be interpreted as a counterfactual mediation analysis or evidence of causal indirect effects (57). Eighth, individual vascular diagnoses and medication records were not available as separate variables in all three datasets, so vascular comorbidity could be represented only by a composite comorbidity index; residual confounding by specific vascular conditions and by their treatment therefore cannot be excluded. Ninth, the proportional-hazards assumption was not fully satisfied for the exposure term; we therefore additionally report period-specific hazard ratios, and the reported hazard ratio should be read as an average effect over follow-up. Finally, although subgroup analyses were exploratory and prespecified, multiple testing and limited precision in smaller subgroups, such as underweight participants in NHANES and some strata in the Second Xiangya Hospital dataset, should be considered when interpreting stratified estimates (29, 30, 58, 59).

## 5 Conclusion

In this study integrating a large prospective UK cohort, a nationally representative US cross-sectional study, and a hospital-based Chinese cross-sectional study, accelerated biological ageing, measured by PhenoAge acceleration, was associated with a higher risk of incident stroke in the UK Biobank and with higher odds of prevalent stroke in NHANES and the Second Xiangya Hospital, with approximately linear exposure-response patterns; because the outcome definitions and temporal structures differ, these estimates are complementary rather than replicative. Exploratory change-in-estimate analyses showed that additional adjustment for healthy-diet adherence reduced these associations only slightly, with absolute changes in the regression coefficient of less than 0.01 on the log scale in every dataset. Subgroup analyses further suggested that the association may be more pronounced among participants with older age, smoking history, long sleep duration, lower income, and poorer diet quality, as well as among socioeconomically disadvantaged participants in datasets where deprivation or poverty-income ratio was available. These subgroup findings should be interpreted cautiously because of multiple comparisons. Whether PhenoAge acceleration has value for clinical risk stratification, and whether improving diet quality would reduce the associated stroke risk, remain to be determined. Prospective studies with repeated measures of ageing biomarkers and diet, together with external validation in clinical populations, are warranted to clarify temporal relationships and potential causal pathways.

## Data Availability

UK Biobank data are available to approved researchers through the UK Biobank access process. NHANES data are publicly available through the National Center for Health Statistics. The de-identified data from the Second Xiangya Hospital are available from the corresponding author upon reasonable request, subject to applicable ethical and institutional requirements. Analytic code or derived materials used for this study are available from the corresponding author upon reasonable request.
