# Supplementary material for: Biological ageing, diet quality, and stroke: evidence from two population-based studies and an exploratory hospital-based Chinese cross-sectional dataset
Source: Front Nutr. 2026 Sep 9;13:1895132. doi: 10.3389/fnut.2026.1895132 (PMC13597419; doi:10.3389/fnut.2026.1895132)
Supplement: Supplementary file 1 [file Supplementary_file_1.docx]

**Biological ageing, diet quality, and stroke: evidence from two population-based studies and an exploratory hospital-based Chinese cross-sectional dataset (Supplementary Materials)**

**Supplementary Texts:**

Supplementary Text S1. Detailed construction of the comorbidity index.

Supplementary Text S2. Detailed classification and explanation of confounders.

Supplementary Text S3. Exclusion of participants who developed stroke within the first three years of follow-up.

Supplementary Text S4. Attenuation analysis after excluding participants who developed stroke within the first three years of follow-up.

Supplementary Text S5. Attenuation analysis with healthy-diet adherence modelled as an ordinal variable.

Supplementary Text S6. Attenuation analysis with BMI modelled as a continuous variable.

Supplementary Text S7. Sensitivity analyses in the Second Xiangya Hospital cross-sectional dataset.

Supplementary Text S8. Dietary questionnaire used in the Second Xiangya Hospital dataset.

**Supplementary Figure:**

**Supplementary Figure S1.** Assumed causal structure relating diet quality, biological ageing and stroke**.**

**Supplementary Tables:**

Table S1. Components and coding rules of the UK Biobank comorbidity index

Table S2. Components and coding rules of the NHANES comorbidity index

Table S3. Classification and explanation of covariates in the UK Biobank and NHANES

Table S4. Association between biological ageing and incident stroke after excluding participants with stroke within the first three years of follow-up

Table S5. Attenuation of the association between PhenoAge acceleration and incident stroke after adjustment for diet quality following exclusion of early stroke events

Table S6. Attenuation of the association between PhenoAge acceleration and incident stroke after adjustment for diet quality when healthy-diet adherence was modelled as an ordinal variable

Table S7. Attenuation of the association between PhenoAge acceleration and incident stroke after adjustment for diet quality when BMI was modelled as a continuous variable

Table S8. Association between biological ageing and stroke in the Second Xiangya Hospital dataset when BMI was modelled as a continuous variable

Table S9. Attenuation of the association between PhenoAge acceleration and stroke after adjustment for diet quality when healthy-diet adherence was modelled as an ordinal variable in the Second Xiangya Hospital dataset

Table S10. Attenuation of the association between PhenoAge acceleration and stroke after adjustment for diet quality when BMI was modelled as a continuous variable in the Second Xiangya Hospital dataset

Table S11. Variable-specific missing data before exclusion, by dataset

Table S12. Comparison of included and excluded participants in the UK Biobank

Table S12b. Comparison of included and excluded eligible adult participants in NHANES

Table S12c. Comparison of included and excluded participants in the Second Xiangya Hospital dataset

Table S13. Correlation of PhenoAge acceleration with chronological age, collinearity diagnostics, and effect estimates under alternative definitions and scalings of the exposure

Table S14. Sequential adjustment models for the association between PhenoAge acceleration and stroke

Table S15. UK Biobank: proportional-hazards testing, incidence rates, stroke subtypes, competing risks and lag analyses

Table S16. Second Xiangya Hospital: sensitivity analyses addressing small-sample bias and overfitting

Table S17. Number of participants and stroke events in each subgroup, UK Biobank and NHANES

Table S18. Exploratory subgroup analyses in the Second Xiangya Hospital dataset, with interaction tests corrected for multiple comparisons

Table S19. Association between diet quality and stroke in the fully adjusted models across the three datasets

**Supplementary Figure S1.** Assumed causal structure relating diet quality, biological ageing and stroke**.** Solid arrows denote assumed causal relations. Diet quality is positioned as an upstream determinant of PhenoAge acceleration and as a shared antecedent of stroke, not as a mediator of the biological ageing–stroke association; comparing models with and without diet quality is therefore a change-in-estimate analysis rather than a mediation analysis. Chronic disease burden lies on the pathway between accelerated biological ageing and stroke, so adjustment for it risks overadjustment. Dashed arrows denote reverse causation, which cannot be excluded in the cross-sectional NHANES and hospital datasets. CKD, chronic kidney disease.

**Supplementary Text S1. Detailed construction of the comorbidity index**

**S1.1 Purpose and general approach**

To account for background chronic disease burden, a comorbidity index was constructed separately in the UK Biobank, NHANES, and the Second Xiangya Hospital dataset. Across datasets, the index was designed to capture background multimorbidity burden before or at the assessment of stroke status or stroke occurrence. The comorbidity index was defined as an unweighted count of chronic conditions, with higher values indicating greater comorbidity burden.

Stroke was excluded from the comorbidity index in all three datasets because it was the outcome of interest. In the UK Biobank, only chronic conditions present at or before baseline were considered eligible for inclusion. In NHANES, the index was based on chronic conditions reported at the survey assessment. Unless otherwise specified, each chronic condition contributed one point to the index if present and zero points if absent. The final comorbidity index was calculated as the sum of all eligible conditions and was analysed as a continuous variable in the main models.

**S1.2 Construction of the comorbidity index in the UK Biobank**

In the UK Biobank, the comorbidity index was derived using baseline self-reported medical history and linked health-record information. Chronic diseases were identified from touchscreen questionnaire responses, nurse-led interview records, hospital inpatient records, and death registry data where available. Conditions were coded as present if the participant reported a physician diagnosis at baseline or if a relevant diagnosis code was recorded at or before baseline. Conditions recorded after baseline were not counted to avoid reverse temporal ordering.

The following chronic conditions were included in the UK Biobank comorbidity index:

1.	Hypertension

2.	Diabetes mellitus

3.	Dyslipidaemia

4.	Coronary heart disease

5.	Heart failure

6.	Atrial fibrillation or other clinically documented arrhythmias

7.	Chronic kidney disease

8.	Chronic liver disease

9.	Chronic lung disease, including chronic bronchitis, emphysema, or chronic obstructive pulmonary disease

10.	Asthma

11.	Malignant tumour or cancer

12.	Arthritis or rheumatic disease

13.	Psychiatric disorder

14.	Memory-related disease or dementia

Each condition was coded as 1 if present and 0 if absent. Repeated records of the same condition were counted only once. The UK Biobank comorbidity index therefore ranged from 0 to 14.

**S1.3 Construction of the comorbidity index in NHANES**

In NHANES, the comorbidity index was constructed using self-reported physician-diagnosed chronic conditions collected during household interviews and health questionnaires. Participants were considered to have a condition if they reported that a doctor or other health professional had ever told them that they had the disease. When laboratory or examination-based information was available and consistent with the disease definition, it was used to complement self-reported information. Stroke was not included in the comorbidity index because it was the outcome variable in NHANES.

The following chronic conditions were included in the NHANES comorbidity index:

1.	Hypertension

2.	Diabetes mellitus

3.	Dyslipidaemia or hypercholesterolaemia

4.	Coronary heart disease

5.	Angina or myocardial infarction

6.	Congestive heart failure

7.	Chronic kidney disease

8.	Chronic liver disease

9.	Chronic lung disease, including chronic bronchitis or emphysema

10.	Asthma

11.	Malignant tumour or cancer

12.	Arthritis

Each condition was coded as 1 if present and 0 if absent. Repeated or overlapping reports of the same disease category were counted only once. The NHANES comorbidity index therefore ranged from 0 to 12.

**S1.4 Construction of the comorbidity index in the Second Xiangya Hospital dataset**

In the Second Xiangya Hospital dataset, the comorbidity index was constructed from physician-documented diagnoses extracted from the electronic medical record at the index clinical encounter. Ten chronic conditions were included: hypertension; diabetes mellitus; dyslipidaemia; coronary heart disease; heart failure; atrial fibrillation; chronic kidney disease; chronic liver disease; chronic lung disease; and malignant tumour. As in the other two datasets, stroke was excluded because it was the outcome of interest. Each condition was coded as 1 if a documented diagnosis was present and 0 if absent, repeated records of the same condition were counted only once, and the index was calculated as the unweighted sum of these conditions, ranging from 0 to 10.

**S1.5 Additional coding rules**

The following coding rules were applied consistently across all three datasets:

1.	Stroke was excluded from the comorbidity index in all participants.

2.	Each chronic disease category contributed a maximum of one point, regardless of the number of repeated records or diagnostic codes.

3.	When multiple related diagnoses referred to the same disease domain, they were harmonized into a single category. For example, myocardial infarction and angina were grouped under coronary heart disease-related conditions where appropriate.

4.	Conditions with uncertain, missing, or ambiguous responses were coded as absent unless a confirmed diagnosis was available from another data source.

5.	In the UK Biobank, conditions recorded after baseline were not counted.

6.	The comorbidity index was treated as a continuous covariate in regression models.

**S1.6 Interpretation and use in the main analyses**

The comorbidity index was used to adjust for baseline multimorbidity burden in the association between biological ageing and stroke. Because accelerated biological ageing may partly reflect cumulative physiological deterioration and chronic disease burden, adjustment for comorbidity was intended to reduce confounding by pre-existing chronic conditions while avoiding adjustment for the stroke outcome itself.

The higher comorbidity index observed among participants with stroke across the three datasets supports the clinical relevance of this variable as an adjustment factor. However, the index was not intended to fully capture disease severity, duration, treatment status, or subclinical disease. Therefore, residual confounding related to chronic disease burden may remain.

**Supplementary Text S2. Detailed classification and explanation of confounders**

This appendix describes the classification and coding of covariates used in the multivariable models for the UK Biobank cohort, NHANES cross-sectional analyses, and the Second Xiangya Hospital cross-sectional analyses. Covariates were selected using an explicit causal framework informed by previous evidence, as described in Section 2.4 of the main text and depicted in Supplementary Figure S1. Where possible, harmonized categories were used across datasets; when identical measurement was not available, dataset-specific variables were used to capture comparable domains.

**S2.1 Harmonization principles**

Most conceptual domains were adjusted for across datasets. When identical variables were unavailable, the closest dataset-specific measure was used. For example, socioeconomic deprivation was captured by the Townsend Deprivation Index in the UK Biobank and by the poverty-income ratio in NHANES. In the Second Xiangya Hospital dataset, race and deprivation or poverty-income ratio were not evaluated, and income level was used as the socioeconomic indicator. Residence was included in the UK Biobank and Second Xiangya Hospital models, but no comparable residence variable was included in the NHANES models.

**S2.2 Treatment of variables in regression models**

Age, sleep duration, BMI, and the comorbidity index were available as continuous variables. In the primary models, BMI and sleep duration were adjusted using categorical forms to allow for potentially non-linear associations with stroke, while comorbidity burden was adjusted as a continuous score. Sensitivity analyses additionally modelled BMI as a continuous variable. Diet quality was not included as a covariate in the base model. A diet-adjusted model additionally included healthy-diet adherence to examine the extent to which the association between PhenoAge acceleration and stroke was attenuated after accounting for diet quality.

**S2.3 Missing or uncertain responses**

Participants with missing information on key exposure, diet quality, outcome, or required covariates were excluded from the analytic sample. For covariates with explicit uncertain response options, such as “prefer not to answer” or “do not know”, values were classified as other/unknown when this category was prespecified; otherwise, they were treated as missing according to the analytic protocol.

**Supplementary Text S3. Exclusion of participants who developed stroke within the first three years of follow-up**

To reduce the possibility of reverse causation, participants who developed stroke within the first three years of follow-up were excluded. Cox proportional hazards models were then refitted using the same covariate-adjustment strategy as the primary analysis. The association between accelerated biological ageing and incident stroke remained statistically significant.

**Supplementary Text S4. Attenuation analysis after excluding participants who developed stroke within the first three years of follow-up**

After excluding participants who developed stroke within the first three years of follow-up, the attenuation analysis was repeated with PhenoAge acceleration as the exposure, healthy-diet adherence as the diet-quality indicator, and incident stroke as the outcome. The attenuation associated with healthy-diet adherence remained statistically significant.

**Supplementary Text S5. Attenuation analysis with healthy-diet adherence modelled as an ordinal variable**

To assess whether the attenuation findings were sensitive to the coding of the diet-quality variable, healthy-diet adherence was alternatively modelled as an ordinal variable. Low, moderate, and high healthy-diet adherence were coded in increasing order, with higher values indicating better diet quality. The results remained materially unchanged.

**Supplementary Text S6. Attenuation analysis with BMI modelled as a continuous variable**

To evaluate whether the results were sensitive to the functional form of BMI adjustment, BMI was modelled as a continuous variable rather than as BMI category. The base model, diet-adjusted model, and attenuation estimate were similar to those observed in the primary attenuation analysis.

**Supplementary Text S7. Sensitivity analyses in the Second Xiangya Hospital cross-sectional dataset**

Because the Second Xiangya Hospital dataset had a cross-sectional design, sensitivity analyses based on exclusion of early incident stroke events were not applicable. Instead, sensitivity analyses focused on alternative model specifications that were relevant to the hospital-based cross-sectional analysis. First, BMI was modelled as a continuous covariate rather than as BMI category. Second, healthy-diet adherence was modelled as an ordinal variable in the attenuation analysis. Third, the attenuation analysis was repeated with BMI entered as a continuous covariate. These analyses used the same covariate-adjustment strategy as the primary Second Xiangya Hospital models, including age, sex, educational level, comorbidity index, smoking status, alcohol consumption, sleep-duration group, income level, and residence. Race and deprivation or poverty-income ratio were not evaluated in this dataset.

The findings were broadly consistent with the primary analysis, although effect estimates were slightly attenuated and confidence intervals were wider than those in the main analysis. When BMI was modelled as a continuous covariate, biologically older participants still had higher odds of stroke than biologically younger participants, and higher quartiles of PhenoAge acceleration remained associated with higher odds of stroke. The attenuation associated with healthy-diet adherence remained modest and statistically significant when healthy-diet adherence was modelled as an ordinal variable and when BMI was modelled continuously, indicating that the exploratory change-in-estimate findings were directionally stable across these specifications in the Second Xiangya Hospital dataset.

**Supplementary Text S8. Dietary questionnaire used in the Second Xiangya Hospital dataset**

Diet quality in the Second Xiangya Hospital dataset was assessed at the index clinical encounter with a structured, interviewer-administered questionnaire covering habitual intake of five major food groups: fruit, vegetables, fish, processed meat and unprocessed red meat. Participants were asked to report usual consumption over the 12 months preceding the index encounter and, for participants with a previous stroke, over the 12 months preceding the stroke event, so that the reported intake refers to habitual pre-stroke diet rather than to any dietary pattern adopted after the event.

Each item was recorded using fixed, closed response categories. Fruit intake and vegetable intake were each recorded on a five-level scale: never or rarely; 1–6 servings per week; 1–2 servings per day; 3–4 servings per day; and 5 or more servings per day. Fish, processed meat and unprocessed red meat intake were each recorded on a six-level scale: never or rarely; 1–3 servings per month; 1 serving per week; 2–3 servings per week; 4–6 servings per week; and 1 or more servings per day. Responses were converted to a five-component Healthy Diet Score using thresholds matched, as closely as the response categories allowed, to those of the UK Biobank Healthy Diet Score: one point was assigned for fruit intake of at least 3 servings per day, vegetable intake of at least 3 servings per day, fish consumption of at least 2 servings per week, processed meat intake of no more than 1 serving per week, and a low intake of unprocessed red meat. The target thresholds were matched as closely as the fixed response categories allowed, and the operational rules below take precedence. The total score ranged from 0 to 5, and participants were categorised as having low (0–1 points), moderate (2–3 points) or high (4–5 points) healthy-diet adherence.

Operationally, one point was assigned when the participant selected the following categories. For fruit and for vegetables, 3–4 servings per day or 5 or more servings per day. For fish, 2–3 servings per week, 4–6 servings per week, or 1 or more servings per day. For processed meat, never or rarely, 1–3 servings per month, or 1 serving per week. For unprocessed red meat, never or rarely, 1–3 servings per month, 1 serving per week, or 2–3 servings per week. Because the response categories were fixed and relatively coarse, a category that straddled the numerical criterion was coded as meeting it; this applied only to unprocessed red meat, for which the operative criterion was therefore no more than 2–3 servings per week rather than the 1–2 servings per week used in the UK Biobank. This is one of several reasons why the hospital score is not equivalent to the UK Biobank Healthy Diet Score, and it is reported here so that the derivation is fully reproducible.

The questionnaire was developed for this study by adapting the UK Biobank dietary items to locally consumed foods and portion descriptions, and it has not been formally validated against weighed dietary records or repeated 24-hour recalls in a Chinese population. Its measurement properties are therefore unknown, and the resulting score is not equivalent to HEI-2020 or to the UK Biobank Healthy Diet Score; this limitation is stated explicitly in the main text.

**Table S1.** Components and coding rules of the UK Biobank comorbidity index

| **Component** | **Coding rule** |
| --- | --- |
| Hypertension | 1 if self-reported or clinically recorded hypertension was present at or before baseline; otherwise 0 |
| Diabetes mellitus | 1 if self-reported or clinically recorded diabetes was present at or before baseline; otherwise 0 |
| Dyslipidaemia | 1 if self-reported or clinically recorded dyslipidaemia or hypercholesterolaemia was present; otherwise 0 |
| Coronary heart disease | 1 if coronary heart disease, myocardial infarction, or angina was recorded; otherwise 0 |
| Heart failure | 1 if heart failure was recorded; otherwise 0 |
| Atrial fibrillation or arrhythmia | 1 if atrial fibrillation or other clinically documented arrhythmia was recorded; otherwise 0 |
| Chronic kidney disease | 1 if chronic kidney disease or renal failure was recorded; otherwise 0 |
| Chronic liver disease | 1 if chronic liver disease was recorded; otherwise 0 |
| Chronic lung disease | 1 if chronic bronchitis, emphysema, or chronic obstructive pulmonary disease was recorded; otherwise 0 |
| Asthma | 1 if asthma was self-reported or clinically recorded; otherwise 0 |
| Malignant tumour | 1 if cancer or malignant tumour was self-reported or clinically recorded; otherwise 0 |
| Arthritis or rheumatic disease | 1 if arthritis, rheumatoid arthritis, osteoarthritis, or rheumatic disease was recorded; otherwise 0 |
| Psychiatric disorder | 1 if depression, anxiety, or other psychiatric disorder was recorded; otherwise 0 |
| Memory-related disease or dementia | 1 if dementia, Alzheimer's disease, or other memory-related disease was recorded; otherwise 0 |

In the analytic UK Biobank sample, the mean comorbidity index was higher among participants who developed incident stroke than among those who remained stroke-free during follow-up. The mean comorbidity index was 1.05 (SD 1.32) among participants without stroke and 1.70 (SD 1.71) among participants with incident stroke.

**Table S2.** Components and coding rules of the NHANES comorbidity index

| **Component** | **Coding rule** |
| --- | --- |
| Hypertension | 1 if physician-diagnosed hypertension was reported, or if hypertension was identified according to examination or medication information where available; otherwise 0 |
| Diabetes mellitus | 1 if physician-diagnosed diabetes was reported, or if diabetes was identified according to laboratory or medication information where available; otherwise 0 |
| Dyslipidaemia or hypercholesterolaemia | 1 if physician-diagnosed high cholesterol or dyslipidaemia was reported, or if lipid-lowering medication use was recorded; otherwise 0 |
| Coronary heart disease | 1 if physician-diagnosed coronary heart disease was reported; otherwise 0 |
| Angina or myocardial infarction | 1 if physician-diagnosed angina or heart attack was reported; otherwise 0 |
| Congestive heart failure | 1 if physician-diagnosed congestive heart failure was reported; otherwise 0 |
| Chronic kidney disease | 1 if weak or failing kidneys, chronic kidney disease, or reduced kidney function was reported or identified; otherwise 0 |
| Chronic liver disease | 1 if physician-diagnosed liver condition was reported; otherwise 0 |
| Chronic lung disease | 1 if chronic bronchitis, emphysema, or chronic obstructive pulmonary disease was reported; otherwise 0 |
| Asthma | 1 if physician-diagnosed asthma was reported; otherwise 0 |
| Malignant tumour | 1 if physician-diagnosed cancer or malignancy was reported; otherwise 0 |
| Arthritis | 1 if physician-diagnosed arthritis was reported; otherwise 0 |

In the analytic NHANES sample, participants with stroke had a higher comorbidity burden than those without stroke. The survey-weighted mean comorbidity index was 0.58 (standard error 0.02) among participants without stroke and 1.35 (standard error 0.05) among participants with self-reported physician-diagnosed stroke, consistent with the weighted estimates presented in Table 1 of the main text.

**Table S3.** Classification and explanation of covariates in the UK Biobank and NHANES

| **Covariate** | **UK Biobank** | **NHANES** |
| --- | --- | --- |
| Age | Age at baseline assessment, analysed as a continuous variable in years. | Age at survey assessment, analysed as a continuous variable in years. |
| Sex | Categorized as male or female. | Categorized as male or female. |
| Race | Categorized as White or non-White. White race included British, Irish, White, and any other White background. Participants reporting other racial or ethnic backgrounds were classified as non-White. | Categorized as White or non-White. Non-Hispanic White participants were classified as White; all other race/ethnicity categories were classified as non-White. |
| Socioeconomic deprivation | Assessed using the Townsend Deprivation Index (TDI). Participants with TDI > 0 were classified as deprived, and those with TDI <= 0 were classified as non-deprived or relatively affluent. | Assessed using the poverty-income ratio (PIR). Lower PIR indicates greater socioeconomic disadvantage. For deprivation analyses, participants in the low PIR group were classified as socioeconomically disadvantaged. |
| Educational level | Categorized as higher, upper secondary, lower secondary, and other/unknown. Higher included college/university degree and other professional qualifications. Upper secondary included A levels/AS levels, NVQ/HND/HNC, or equivalent qualifications. Lower secondary included O levels/GCSEs, CSEs, or equivalent qualifications. Other/unknown included none of the above, prefer not to answer, or missing/uncertain responses. | Categorized as higher, upper secondary, lower secondary, and other/unknown. Higher included college graduate or above. Upper secondary included some college or associate degree. Lower secondary included high school graduate/GED or equivalent. Other/unknown included less than high school, no diploma, refused, do not know, or missing/uncertain responses. |
| Body mass index | BMI was calculated as weight in kilograms divided by height in meters squared and analysed as both a continuous and categorical variable. Categories were: underweight, BMI <18.5 kg/m²; normal weight, 18.5 <= BMI <25 kg/m²; overweight, 25 <= BMI <30 kg/m²; obesity, BMI >=30 kg/m². | BMI was calculated as weight in kilograms divided by height in meters squared and analysed as both a continuous and categorical variable. Categories were: underweight, BMI <18.5 kg/m²; normal weight, 18.5 <= BMI <25 kg/m²; overweight, 25 <= BMI <30 kg/m²; obesity, BMI >=30 kg/m². |
| Comorbidity index | An unweighted count of pre-existing chronic diseases, analysed as a continuous variable. The detailed components and coding rules are provided in Supplementary materials 1. | An unweighted count of physician-diagnosed chronic diseases, analysed as a continuous variable. The detailed components and coding rules are provided in Supplementary materials 1. |
| Smoking status | Assessed using the question on ever smoking cigarettes. Categorized as never smoking or ever smoking. | Assessed using self-reported cigarette-smoking history. Categorized as never smoking or ever smoking. |
| Alcohol consumption | Categorized as never, previous, current, or unknown alcohol consumption. | Categorized as never, previous, current, or unknown alcohol consumption according to survey responses. |
| Sleep duration | Self-reported average sleep duration per day, analysed as a continuous variable in hours and as a categorical variable. Categories were: short sleep, <7 hours/day; optimal sleep, 7 to 9 hours/day; long sleep, >9 hours/day. | Self-reported average sleep duration per day, analysed as a continuous variable in hours and as a categorical variable. Categories were: short sleep, <7 hours/day; optimal sleep, 7 to 9 hours/day; long sleep, >9 hours/day. |
| Income or PIR | Annual household income before tax was categorized as low, middle, and high. Low income included <£31,000; middle income included £31,000 to £100,000; high income included >£100,000. | Socioeconomic level was categorized according to PIR as low, middle, and high. Low PIR was defined as PIR <1.30, middle PIR as 1.30 <= PIR <3.50, and high PIR as PIR >=3.50. |
| Residence | Categorized as urban, rural, or unknown according to residential classification data. | - |

**Table S4.** Association between biological ageing and incident stroke after excluding participants with stroke within the first three years of follow-up

| **Variable** | **Comparison** | **HR (95% CI)** | ***P*-value** |
| --- | --- | --- | --- |
| Biological ageing status | Biologically younger | 1.000 (Reference) |  |
|  | Biologically older | 1.701 (1.634-1.772) | <0.001 |
| PhenoAge acceleration quartiles | Quartile 1 | 1.000 (Reference) |  |
|  | Quartile 2 | 1.361 (1.267-1.462) | <0.001 |
|  | Quartile 3 | 1.828 (1.698-1.968) | <0.001 |
|  | Quartile 4 | 2.704 (2.503-2.921) | <0.001 |
| PhenoAge acceleration | Per 1-year increase | 1.058 (1.054-1.062) | <0.001 |

Note: HR, hazard ratio; CI, confidence interval; PhenoAge, phenotypic age. This sensitivity analysis was restricted to the UK Biobank cohort. Models were adjusted for age, sex, race, educational level, BMI category, comorbidity index, smoking status, alcohol consumption, sleep-duration group, Townsend Deprivation Index, income level, and residence. Diet quality was not included in this base model; the diet-adjusted model was evaluated separately in the attenuation analysis.

**Table S5.** Attenuation of the association between PhenoAge acceleration and incident stroke after adjustment for diet quality following exclusion of early stroke events

| **Effect estimate** | **Scale** | **Estimate (95% CI)** | ***P*-value** |
| --- | --- | --- | --- |
| Base model | HR | 1.058 (1.054-1.062) | <0.001 |
| Diet-adjusted model | HR | 1.052 (1.048-1.056) | <0.001 |
| Attenuation estimate associated with diet quality | HR-scale | 1.006 (1.004-1.009) | <0.001 |
| Percentage attenuation | % | 11.2% (7.9%-14.9%) | <0.001 |

CI, confidence interval; HR, hazard ratio. The base model was estimated without adjustment for healthy-diet adherence, whereas the diet-adjusted model additionally adjusted for healthy-diet adherence. The attenuation estimate was calculated on the log-risk scale. Confidence intervals were obtained using bootstrap resampling. All models used the UK Biobank covariate-adjustment strategy, including age, sex, race, educational level, BMI category, comorbidity index, smoking status, alcohol consumption, sleep-duration group, Townsend Deprivation Index, income level, and residence.

**Table S6.** Attenuation of the association between PhenoAge acceleration and incident stroke after adjustment for diet quality when healthy-diet adherence was modelled as an ordinal variable

| **Effect estimate** | **Scale** | **Estimate (95% CI)** | ***P*-value** |
| --- | --- | --- | --- |
| Base model | HR | 1.060 (1.056-1.064) | <0.001 |
| Diet-adjusted model | HR | 1.054 (1.050-1.058) | <0.001 |
| Attenuation estimate associated with diet quality | HR-scale | 1.006 (1.004-1.008) | <0.001 |
| Percentage attenuation | % | 10.7% (7.5%-14.3%) | <0.001 |

CI, confidence interval; HR, hazard ratio. Healthy-diet adherence was entered as an ordinal diet-quality variable. The base model, diet-adjusted model, attenuation estimate, and percentage attenuation were estimated using the same UK Biobank covariate-adjustment strategy as the primary attenuation analysis.

**Table S7.** Attenuation of the association between PhenoAge acceleration and incident stroke after adjustment for diet quality when BMI was modelled as a continuous variable

| **Effect estimate** | **Scale** | **Estimate (95% CI)** | ***P*-value** |
| --- | --- | --- | --- |
| Base model | HR | 1.059 (1.055-1.063) | <0.001 |
| Diet-adjusted model | HR | 1.052 (1.048-1.056) | <0.001 |
| Attenuation estimate associated with diet quality | HR-scale | 1.007 (1.005-1.009) | <0.001 |
| Percentage attenuation | % | 11.6% (8.2%-15.2%) | <0.001 |

BMI, body mass index; CI, confidence interval; HR, hazard ratio. BMI was entered as a continuous covariate in kg/m^2^. All other covariates were specified as in the UK Biobank primary attenuation analysis.

**Table S8.** Association between biological ageing and stroke in the Second Xiangya Hospital dataset when BMI was modelled as a continuous variable

| **Variable** | **Comparison** | **OR (95% CI)** | ***P*-value** |
| --- | --- | --- | --- |
| Biological ageing status | Biologically younger | 1.000 (Reference) |  |
|  | Biologically older | 1.742 (1.071-2.833) | 0.026 |
| PhenoAge acceleration quartiles | Quartile 1 | 1.000 (Reference) |  |
|  | Quartile 2 | 1.198 (0.762-1.883) | 0.431 |
|  | Quartile 3 | 1.641 (1.003-2.686) | 0.049 |
|  | Quartile 4 | 1.901 (1.108-3.262) | 0.020 |
| PhenoAge acceleration | Per 1-year increase | 1.049 (1.009-1.091) | 0.016 |

OR, odds ratio; CI, confidence interval; PhenoAge, phenotypic age; BMI, body mass index. This sensitivity analysis was restricted to the Second Xiangya Hospital cross-sectional dataset. BMI was entered as a continuous covariate in kg/m^2^. Models were adjusted for age, sex, educational level, continuous BMI, comorbidity index, smoking status, alcohol consumption, sleep-duration group, income level, and residence. Diet quality was not included in this base model; the diet-adjusted model was evaluated separately in the attenuation analysis.

**Table S9.** Attenuation of the association between PhenoAge acceleration and stroke after adjustment for diet quality when healthy-diet adherence was modelled as an ordinal variable in the Second Xiangya Hospital dataset

| **Effect estimate** | **Scale** | **Estimate (95% CI)** | ***P*-value** |
| --- | --- | --- | --- |
| Base model | OR | 1.078 (1.003-1.158) | 0.041 |
| Diet-adjusted model | OR | 1.074 (1.000-1.153) | 0.049 |
| Attenuation estimate associated with diet quality | OR-scale | 1.004 (1.001-1.008) | 0.047 |
| Percentage attenuation | % | 4.8% (0.2%-10.3%) | 0.046 |

CI, confidence interval; OR, odds ratio. Healthy-diet adherence was entered as an ordinal diet-quality variable, with low, moderate, and high adherence coded in increasing order. The base model was estimated without adjustment for healthy-diet adherence, whereas the diet-adjusted model additionally adjusted for healthy-diet adherence. The attenuation estimate was calculated on the log-odds scale, and confidence intervals were obtained using bootstrap resampling. Models used the Second Xiangya Hospital covariate-adjustment strategy, including age, sex, educational level, BMI category, comorbidity index, smoking status, alcohol consumption, sleep-duration group, income level, and residence.

**Table S10.** Attenuation of the association between PhenoAge acceleration and stroke after adjustment for diet quality when BMI was modelled as a continuous variable in the Second Xiangya Hospital dataset

| **Effect estimate** | **Scale** | **Estimate (95% CI)** | ***P*-value** |
| --- | --- | --- | --- |
| Base model | OR | 1.081 (1.006-1.163) | 0.034 |
| Diet-adjusted model | OR | 1.076 (1.002-1.156) | 0.044 |
| Attenuation estimate associated with diet quality | OR-scale | 1.005 (1.001-1.009) | 0.039 |
| Percentage attenuation | % | 5.7% (0.5%-11.5%) | 0.034 |

BMI, body mass index; CI, confidence interval; OR, odds ratio. BMI was entered as a continuous covariate in kg/m^2^. The base model was estimated without adjustment for healthy-diet adherence, whereas the diet-adjusted model additionally adjusted for healthy-diet adherence. The attenuation estimate was calculated on the log-odds scale, and confidence intervals were obtained using bootstrap resampling. All other covariates were specified as in the primary Second Xiangya Hospital attenuation analysis.

**Table S11.** Variable-specific missing data before exclusion, by dataset

| **Variable** | **UK Biobank n (%)** | **NHANES n (%)** | **Second Xiangya Hospital n (%)** |
| --- | --- | --- | --- |
| **PhenoAge biomarker panel (all nine components)** | **90,653 (18.37)** | **14,832 (15.47)** | **19 (4.86)** |
| **Individual PhenoAge biomarkers** |  |  |  |
| Serum albumin | 32,541 (6.59) | 3,214 (3.35) | 3 (0.77) |
| Creatinine | 28,154 (5.70) | 2,951 (3.08) | 2 (0.51) |
| Glucose | 65,412 (13.25) | 8,142 (8.49) | 8 (2.05) |
| C-reactive protein | 72,105 (14.61) | 12,540 (13.08) | 14 (3.58) |
| Lymphocyte percentage | 35,410 (7.17) | 4,102 (4.28) | 5 (1.28) |
| Mean corpuscular volume | 35,214 (7.13) | 4,055 (4.23) | 4 (1.02) |
| Red cell distribution width | 35,320 (7.16) | 4,120 (4.30) | 4 (1.02) |
| Alkaline phosphatase | 30,145 (6.11) | 3,514 (3.66) | 6 (1.53) |
| White blood cell count | 35,112 (7.11) | 4,015 (4.19) | 4 (1.02) |
| Diet-quality score | 8,192 (1.66) | 5,124 (5.34) | 15 (3.84) |
| Body mass index | 9,595 (1.94) | 1,489 (1.55) | 0 (0.00) |
| Educational level | 9,906 (2.01) | 1,123 (1.17) | 6 (1.53) |
| Comorbidity index | 12,515 (2.54) | 2,341 (2.44) | 0 (0.00) |
| Smoking status | 2,892 (0.59) | 1,309 (1.37) | 0 (0.00) |
| Alcohol consumption | 1,617 (0.33) | 1,765 (1.84) | 0 (0.00) |
| Sleep duration | 875 (0.18) | 2,014 (2.10) | 0 (0.00) |
| Townsend Deprivation Index / PIR | 636 (0.13) | 8,112 (8.46) | Not evaluated |
| Race or ethnicity | 2,726 (0.55) | 903 (0.94) | Not evaluated |
| Income level | 75,443 (15.29) | 8,437 (8.80) | 0 (0.00) |
| Residence | 4,983 (1.01) | Not evaluated | 3 (0.77) |

Denominators are the eligible participants remaining after exclusion of prevalent stroke at or before baseline (UK Biobank, n = 493,546), all NHANES participants screened (n = 95,877) and all participants screened for the hospital dataset (n = 391). PIR, poverty-income ratio. Percentages are column percentages of the corresponding denominator. Because the PhenoAge algorithm requires all nine biomarkers, missingness for the complete panel reflects participants missing at least one component and therefore exceeds the missingness of any single biomarker. In NHANES, missingness of C-reactive protein varied systematically across survey cycles because the biomarker was not assessed in the 2011–2012 and 2013–2014 cycles, which accounts for the higher missingness of this component; missingness of the remaining biomarkers did not show a clear systematic pattern across cycles.

**Table S12a.** Comparison of included and excluded participants in the UK Biobank

| Characteristic | Included (n = 400,695) | Excluded (n = 92,851) | SMD |
| --- | --- | --- | --- |
| Age, years, mean (SD) | 56.13 (8.06) | 56.32 (8.15) | 0.023 |
| Body mass index, kg/m2, mean (SD) | 27.37 (4.73) | 27.49 (4.89) | 0.025 |
| Townsend Deprivation Index, mean (SD) | -1.35 (3.06) | -1.05 (3.22) | 0.095 |
| Comorbidity index, mean (SD) | 1.07 (1.34) | 1.25 (2.05) | 0.103 |
| Sleep duration, hours, mean (SD) | 7.15 (1.07) | 7.02 (1.50) | 0.100 |
| Male sex, % | 47.1 | 43.6 | 0.070 |
| White ethnicity, % | 95.2 | 92.5 | 0.106 |
| Higher education, % | 46.9 | 44.0 | 0.058 |
| Ever smoking, % | 45.1 | 44.8 | 0.006 |
| Current alcohol use, % | 92.9 | 90.8 | 0.073 |
| High healthy-diet adherence, % | 34.9 | 36.1 | 0.025 |
| Any recorded stroke diagnosis, % | 2.83 | 2.88 | 0.003 |

SMD, standardised mean difference. All standardised mean differences were below 0.11, indicating that participants included in the complete-case analysis were closely comparable with those excluded, and that selection bias arising from complete-case analysis is unlikely to be material.

**Table S12b. Comparison of included and excluded eligible adult participants in NHANES**

| **Characteristic** | **Included (n = 21,754)** | **Excluded (n = 22,195)** | **SMD** |
| --- | --- | --- | --- |
| Age, years, mean (SD) | 46.51 (16.20) | 44.82 (17.51) | 0.100 |
| Body mass index, kg/m2, mean (SD) | 28.65 (6.54) | 28.21 (6.88) | 0.065 |
| Poverty-income ratio, mean (SD) | 2.84 (1.52) | 2.61 (1.61) | 0.146 |
| Comorbidity index, mean (SD) | 0.61 (0.72) | 0.54 (0.69) | 0.099 |
| Male sex, % | 48.7 | 49.2 | 0.010 |
| White race, % | 40.9 | 38.6 | 0.047 |
| Higher education, % | 28.5 | 25.2 | 0.074 |
| Ever smoking, % | 43.4 | 42.1 | 0.026 |
| Self-reported stroke history, % | 3.35 | 3.20 | 0.008 |

SMD, standardised mean difference. The comparison was restricted to eligible adults (aged 18 years or older and not pregnant) so that complete-case bias could be assessed meaningfully. Standardised mean differences were all below 0.15, although excluded participants were slightly younger and had a slightly lower mean poverty-income ratio, which is the pattern usually seen with non-response in health examination surveys.

**Table S12c. Comparison of included and excluded participants in the Second Xiangya Hospital dataset**

| **Characteristic** | **Included (n = 312)** | **Excluded (n = 79)** | **SMD** |
| --- | --- | --- | --- |
| Age, years, mean (SD) | 59.34 (12.82) | 60.15 (13.55) | 0.061 |
| Body mass index, kg/m2, mean (SD) | 25.89 (3.73) | 26.21 (4.01) | 0.082 |
| Comorbidity index, mean (SD) | 0.99 (0.83) | 1.10 (0.95) | 0.123 |
| Male sex, % | 51.9 | 53.2 | 0.026 |
| Higher education, % | 27.8 | 24.1 | 0.085 |
| Ever smoking, % | 39.1 | 41.7 | 0.053 |
| Current alcohol use, % | 69.2 | 67.1 | 0.045 |
| Physician-diagnosed stroke, % | 18.59 | 21.52 | 0.073 |

SMD, standardised mean difference. Excluded participants (n = 79) were those screened but missing complete PhenoAge biomarkers, a diet-quality score, or essential covariates. All standardised mean differences were below 0.15, indicating that the analytic sample remained broadly comparable with the screened clinical source population.

**Table S13.** Correlation of PhenoAge acceleration with chronological age, collinearity diagnostics, and effect estimates under alternative definitions and scalings of the exposure

| **Quantity** | **UK Biobank** | **NHANES** | **Second Xiangya Hospital** |
| --- | --- | --- | --- |
| Pearson r (chronological age, PhenoAge) | 0.878 | 0.824 | 0.616 |
| Pearson r (chronological age, PhenoAge acceleration) | -0.021 | -0.083 | 0.108 |
| Pearson r (chronological age, residual-based acceleration) | 0.000 | 0.000 | 0.000 |
| SD of PhenoAge acceleration, years | 4.36 | 4.62 | 4.31 |
| Maximum VIF in the primary model | 1.11 | 1.34 | 1.95 |
| VIF for PhenoAge acceleration | 1.07 | 1.12 | 1.05 |
| Primary estimate, per 1-year increase | 1.060 (1.056-1.064) | 1.058 (1.037-1.080) | 1.082 (1.002-1.169) |
| Per 1 SD increase | 1.291 (1.270-1.313) | 1.299 (1.182-1.428) | 1.406 (1.008-1.961) |
| Per 5-year increase | 1.338 (1.313-1.364) | Not applicable | Not applicable |
| Residual-based acceleration, per 1 year | 1.059 (1.055-1.063) | 1.055 (1.034-1.077) | 1.067 (1.031-1.105) |
| Chronological age as restricted cubic spline | 1.057 (1.053-1.061) | 1.061 (1.040-1.083) | 1.081 (1.000-1.168) |

Estimates are hazard ratios (UK Biobank) or odds ratios (NHANES and Second Xiangya Hospital) with 95% confidence intervals, from models adjusted for the covariates of the corresponding primary model. Variance inflation factors are generalised variance inflation factors expressed as GVIF^(1/2df) for multi-category terms. The residual-based definition regresses PhenoAge on chronological age within each dataset and is orthogonal to chronological age by construction. The near-zero correlation between chronological age and PhenoAge acceleration in the UK Biobank, together with variance inflation factors close to unity in all datasets, indicates that simultaneous adjustment for chronological age does not induce material collinearity. SD, standard deviation; VIF, variance inflation factor.

**Table S14.** Sequential adjustment models for the association between PhenoAge acceleration and stroke

| **Model** | **UK Biobank** | **NHANES** | **Second Xiangya Hospital** |
| --- | --- | --- | --- |
| Model 1: chronological age and sex | 1.084 (1.080-1.088) | 1.088 (1.067-1.109) | 1.080 (1.007-1.158) |
| Model 2: + sociodemographic and lifestyle variables | 1.077 (1.073-1.081) | 1.075 (1.054-1.096) | 1.078 (1.000-1.163) |
| Model 3: + comorbidity burden | 1.060 (1.056-1.064) | 1.058 (1.037-1.080) | 1.082 (1.002-1.169) |
| Model 4: + diet quality | 1.053 (1.049-1.057) | 1.052 (1.031-1.074) | 1.081 (1.001-1.168) |
| Excluding potentially downstream comorbidities | 1.079 (1.075-1.083) | 1.078 (1.057-1.099) | 1.078 (1.000-1.163) |

Estimates are hazard ratios (UK Biobank) or odds ratios (NHANES and Second Xiangya Hospital) per 1-year increase in PhenoAge acceleration, with 95% confidence intervals. Model 2 adds race or ethnicity where available, educational level, body mass index category, smoking status, alcohol consumption, sleep-duration group, socioeconomic deprivation and residence. Sequential estimates are stable across all four models in every dataset, indicating that the association is not generated by any single adjustment step. Individual vascular diagnoses and medication records were not available as separate variables in all three datasets, so vascular comorbidity is represented by the composite comorbidity index.

**Table S15.** UK Biobank: proportional-hazards testing, incidence rates, stroke subtypes, competing risks and lag analyses

| **Analysis** | **Events** | **Estimate (95% CI)** | **Test statistic** | |
| --- | --- | --- | --- | --- |
| Proportional hazards, exposure term | - | - | χ² = 13.83, df = 1, P = 2.0×10⁻⁴ |  |
| Proportional hazards, global test | - | - | χ² = 209.63, df = 27, P < 0.001 | |
| Incidence rate, biologically younger, per 1,000 person-years | 8,044 | 2.00 per 1,000 person-years | - | |
| Incidence rate, biologically older, per 1,000 person-years | 3,301 | 3.01 per 1,000 person-years | - | |
| Ischaemic stroke (I63) | 7,459 | HR 1.066 (1.061-1.071) | - | |
| Haemorrhagic stroke (I60-I62) | 3,886 | HR 1.009 (0.998-1.020) | - | |
| Cause-specific hazard model for stroke (identical to the primary Cox model, in which death is a censoring event) | 11,345 | HR 1.060 (1.056-1.064) | - | |
| Fine-Gray subdistribution hazard model | 11,345 | sHR 1.041 (1.037-1.045) | - | |
| Excluding events within 3 years | 10,211 | HR 1.058 (1.054-1.062) | - | |
| Excluding events within 5 years | 9,034 | HR 1.055 (1.051-1.059) | - | |
| Excluding events within 7 years | 7,852 | HR 1.052 (1.048-1.056) | - | |
| Censored at the earliest national linkage date (28 February 2018) | 8,942 | HR 1.059 (1.055-1.063) | - | |
| Period-specific hazard ratio, 0-5 years | 2,311 | HR 1.071 (1.062-1.080) | - | |
| Period-specific hazard ratio, 5-10 years | 3,458 | HR 1.063 (1.056-1.070) | - | |
| Period-specific hazard ratio, >10 years | 5,576 | HR 1.051 (1.046-1.056) | - | |

Hospital inpatient linkage was complete to 31 October 2022 in England, 31 July 2021 in Scotland and 28 February 2018 in Wales; follow-up was censored at the nation-specific linkage end date. These three dates were confirmed directly from the distribution of censoring dates in the analytic data. Stroke subtypes were defined to be mutually exclusive. Events carrying diagnostic codes for both ischaemic (I63) and haemorrhagic (I60–I62) stroke were classified according to the primary recorded cause. Events coded strictly as stroke not specified as haemorrhage or infarction (I64), without any concurrent specific subtype code, were excluded from the subtype-specific analyses; no such event occurred in the analytic sample, so the two subtype counts sum to the total number of stroke events. In the cause-specific framework, censoring at death is the appropriate treatment of the competing event, so the primary Cox model already provides the cause-specific hazard estimate and the two are identical by construction. The Fine–Gray subdistribution hazard model is reported as a complementary analysis on the cumulative-incidence scale, with death before stroke as the competing event. Because the proportional-hazards assumption was not satisfied for the exposure term, period-specific hazard ratios are reported and the overall hazard ratio should be interpreted as an average effect over follow-up.

**Table S16.** Second Xiangya Hospital: sensitivity analyses addressing small-sample bias and overfitting

| **Analysis** | **Parameters** | **Events per parameter** | **OR (95% CI)** | ***P*** |
| --- | --- | --- | --- | --- |
| Fully adjusted model (primary) | 18 | 3.2 | 1.082 (1.002-1.169) | 0.045 |
| Parsimonious core adjustment set | 7 | 8.3 | 1.082 (1.007-1.163) | 0.031 |
| Firth penalised logistic regression, fully adjusted | 18 | 3.2 | 1.074 (0.998-1.157) | 0.056 |
| Firth penalised logistic regression, core set | 7 | 8.3 | 1.080 (1.006-1.160) | 0.033 |
| Bootstrap, 2,000 resamples (median, percentile CI) | 18 | 3.2 | 1.086 (0.993-1.208) | - |
| Average marginal effect, base model | - | - | 0.00957 | - |
| Average marginal effect, diet-adjusted model | - | - | 0.00947 | - |

The parsimonious core adjustment set comprised chronological age, sex, body mass index, comorbidity burden and smoking status, selected on causal grounds rather than by statistical screening. Across 2,000 bootstrap resamples the coefficient for PhenoAge acceleration was positive in 96.4% of replications; the percentile interval nevertheless included the null, so the direction of the association is stable but its statistical significance is fragile. Average marginal effects are absolute risk differences per 1-year increase in PhenoAge acceleration and are reported because, unlike the odds ratio, they are collapsible and therefore comparable across nested models; on this scale additional adjustment for diet quality changed the estimate by 0.00009, approximately 1.0% of the base-model effect. OR, odds ratio; CI, confidence interval. All logistic-regression specifications reported in this table converged.

**Table S16 (continued).** Estimates after multiple imputation and after inverse-probability-of-completeness weighting

| Analysis (per 1-year increase) | UK Biobank (HR, 95% CI) | NHANES (OR, 95% CI) | Second Xiangya Hospital (OR, 95% CI) |  |
| --- | --- | --- | --- | --- |
| Multiple imputation (m = 20) | 1.058 (1.054-1.062) | 1.056 (1.036-1.077) | 1.077 (1.000-1.159) |  |
| Inverse-probability-of-completeness weighting | 1.059 (1.055-1.063) | 1.055 (1.034-1.076) | 1.081 (1.001-1.167) |  |

**Table S17.** Number of participants and stroke events in each subgroup, UK Biobank and NHANES

| **Subgroup variable** | **UK Biobank n / events** | **NHANES n / events** |
| --- | --- | --- |
| **Age group** |  |  |
| <60 years | 261,842 / 5,118 | 14,580 / 112 |
| ≥60 years | 138,853 / 6,227 | 7,174 / 617 |
| **Sex** |  |  |
| Female | 211,888 / 4,936 | 11,266 / 399 |
| Male | 188,807 / 6,409 | 10,488 / 330 |
| **Race or ethnicity** |  |  |
| White | 381,407 / 10,859 | 8,910 / 332 |
| Non-White | 19,288 / 486 | 12,844 / 397 |
| **Educational level** |  |  |
| Higher | 188,123 / 4,757 | 6,208 / 112 |
| Upper secondary | 132,102 / 3,495 | 7,055 / 201 |
| Lower secondary | 78,935 / 3,028 | 8,336 / 409 |
| **BMI category** |  |  |
| Underweight | 2,793 / 68 | 365 / 7 |
| Normal weight | 130,614 / 2,916 | 6,048 / 161 |
| Overweight | 172,186 / 4,765 | 6,971 / 243 |
| Obesity | 95,102 / 3,596 | 8,370 / 318 |
| **Comorbidity index** |  |  |
| Index 0 | 180,452 / 2,105 | 12,504 / 144 |
| Index 1-2 | 155,830 / 4,562 | 6,412 / 281 |
| Index ≥3 | 64,413 / 4,678 | 2,838 / 304 |
| **Smoking status** |  |  |
| Never | 219,828 / 5,354 | 12,311 / 294 |
| Ever | 180,867 / 5,991 | 9,443 / 435 |
| **Alcohol consumption** |  |  |
| Never or previous | 28,174 / 998 | 6,833 / 319 |
| Current | 372,300 / 10,340 | 14,921 / 410 |
| **Sleep-duration group** |  |  |
| Optimal | 297,720 / 8,181 | 13,172 / 391 |
| Short | 96,523 / 2,883 | 7,267 / 260 |
| Long | 6,452 / 281 | 1,315 / 78 |
| **Deprivation or poverty-income ratio** |  |  |
| Non-deprived or high PIR | 289,845 / 7,893 | 14,917 / 424 |
| Deprived or low PIR | 110,850 / 3,452 | 6,837 / 305 |
| **Income level** |  |  |
| Low | 189,943 / 6,863 | 6,837 / 305 |
| Middle | 188,696 / 4,121 | 8,156 / 295 |
| High | 22,056 / 361 | 6,761 / 129 |
| **Healthy-diet adherence** |  |  |
| Low | 19,378 / 680 | 5,703 / 236 |
| Moderate | 241,294 / 6,900 | 12,002 / 396 |
| High | 140,023 / 3,765 | 4,049 / 97 |
| **Residence** |  |  |
| Urban | 345,041 / 9,795 | Not evaluated |
| Rural | 55,648 / 1,550 | Not evaluated |

Interaction *P* values are reported in the corresponding forest plots (Figure 3) and are additionally presented after Benjamini-Hochberg correction for multiple comparisons. Entries are the number of participants and the number of stroke events in each stratum. Categories coded as other or unknown are not shown separately, so for educational level, alcohol consumption and residence the strata sum to slightly fewer than the full analytic sample. Residence was not evaluated in NHANES.

**Table S18.** Exploratory subgroup analyses in the Second Xiangya Hospital dataset, with interaction tests corrected for multiple comparisons

| **Subgroup** | **n** | **Events** | **OR (95% CI)** | ***P* for interaction** | **BH-adjusted q** |
| --- | --- | --- | --- | --- | --- |
| Age group |  |  |  | 0.266 | 0.665 |
| <60 years | 158 | 17 | 1.28 (0.78-2.11) |  |  |
| ≥60 years | 154 | 41 | 1.86 (1.20-2.88) |  |  |
| Sex |  |  |  | 0.495 | 0.952 |
| Female | 150 | 23 | 1.54 (1.01-2.36) |  |  |
| Male | 162 | 35 | 1.63 (1.05-2.55) |  |  |
| Educational level |  |  |  | 0.952 | 0.952 |
| Higher | 87 | 9 | 1.31 (0.73-2.35) |  |  |
| Upper secondary | 101 | 16 | 1.44 (0.85-2.44) |  |  |
| Lower secondary | 118 | 31 | 1.83 (1.16-2.91) |  |  |
| Other/unknown | 6 | 2 | 1.55 (0.41-5.85) |  |  |
| BMI category |  |  |  | 0.145 | 0.665 |
| Underweight | 9 | 1 | 1.42 (0.32-6.80) |  |  |
| Normal weight | 104 | 14 | 1.36 (0.72-2.59) |  |  |
| Overweight | 122 | 22 | 1.57 (1.01-2.46) |  |  |
| Obesity | 77 | 21 | 1.75 (1.04-2.94) |  |  |
| Smoking status |  |  |  | 0.200 | 0.665 |
| Never | 190 | 27 | 1.38 (0.83-2.30) |  |  |
| Ever | 122 | 31 | 1.87 (1.21-2.89) |  |  |
| Alcohol consumption |  |  |  | 0.656 | 0.952 |
| Never | 55 | 15 | 1.65 (0.88-3.08) |  |  |
| Previous | 41 | 13 | 1.78 (0.92-3.44) |  |  |
| Current | 216 | 30 | 1.44 (0.94-2.22) |  |  |
| Sleep-duration group |  |  |  | 0.242 | 0.665 |
| Optimal sleep | 200 | 31 | 1.42 (0.92-2.19) |  |  |
| Short | 89 | 18 | 1.62 (0.97-2.72) |  |  |
| Long | 23 | 9 | 1.91 (1.04-3.50) |  |  |
| Income level |  |  |  | 0.841 | 0.952 |
| Low | 103 | 28 | 1.89 (1.21-2.95) |  |  |
| Middle | 137 | 23 | 1.47 (0.94-2.30) |  |  |
| High | 72 | 7 | 1.29 (0.64-2.58) |  |  |
| Residence |  |  |  | 0.946 | 0.952 |
| Urban | 214 | 33 | 1.72 (0.91-3.18) |  |  |
| Rural | 95 | 24 | 1.76 (0.83-3.73) |  |  |
| Healthy-diet adherence |  |  |  | 0.943 | 0.952 |
| Low adherence | 85 | 24 | 1.84 (1.12-3.03) |  |  |
| Moderate adherence | 166 | 28 | 1.51 (0.97-2.35) |  |  |
| High adherence | 61 | 6 | 1.34 (0.58-3.07) |  |  |

Odds ratios compare biologically older with biologically younger participants within each stratum, from the fully adjusted models. Interaction *P* values are from likelihood ratio tests of the product term in the fully adjusted model and were corrected across the ten tests using the Benjamini-Hochberg false-discovery-rate procedure. The smallest unadjusted *P* value was 0.145 and the smallest adjusted q value was 0.665; no interaction was significant after correction. All strata were fitted with the same fully adjusted covariate set used in the primary hospital-based model. These analyses are exploratory and, given 58 events in total, the stratum-specific estimates are imprecise and should not be interpreted as evidence of effect modification. BH, Benjamini-Hochberg; BMI, body mass index; OR, odds ratio; CI, confidence interval. Residence was not recorded for 3 participants, 1 of whom had stroke; these participants are therefore not shown as a separate stratum, so the residence strata sum to fewer than the full analytic sample.

**Table S19. Association between diet quality and stroke in the fully adjusted models across the three datasets**

| **Dataset** | **Diet-quality category** | **Estimate (95% CI)** | ***P*-value** |
| --- | --- | --- | --- |
| UK Biobank (hazard ratios) | Low adherence | 1.000 (reference) | - |
|  | Moderate adherence | 0.985 (0.931-1.042) | 0.601 |
|  | High adherence | 0.962 (0.908-1.019) | 0.188 |
| NHANES (odds ratios) | Low adherence | 1.000 (reference) | - |
|  | Moderate adherence | 0.951 (0.835-1.082) | 0.448 |
|  | High adherence | 0.914 (0.785-1.065) | 0.252 |
| Second Xiangya Hospital (odds ratios) | Low adherence | 1.000 (reference) | - |
|  | Moderate adherence | 0.885 (0.435-1.802) | 0.736 |
|  | High adherence | 0.762 (0.285-2.038) | 0.589 |

Estimates are from the fully adjusted models without PhenoAge acceleration, adjusted for chronological age, sex, race or ethnicity where available, educational level, body mass index category, comorbidity index, smoking status, alcohol consumption, sleep-duration group and the dataset-specific socioeconomic indicator (Townsend Deprivation Index, poverty-income ratio or income level). In all three datasets the direct association between diet quality and stroke was not statistically significant after adjustment for these covariates. CI, confidence interval.
